# Human CD4^+^ T cells recognize *Mycobacterium tuberculosis*–infected macrophages amid broader responses

**DOI:** 10.1084/jem.20250460

**Published:** 2025-09-24

**Authors:** Volodymyr Stetsenko, Daniel P. Gail, Scott M. Reba, Vinicius G. Suzart, Robert Schauner, Avinaash K. Sandhu, Alessandro Sette, Mohammad Haj Dezfulian, Cecilia S. Lindestam Arlehamn, Stephen M. Carpenter

**Affiliations:** 1 Division of Infectious Diseases and HIV Medicine, Department of Medicine, University Hospitals Cleveland Medical Center, Case Western Reserve University School of Medicine, Cleveland, OH, USA; 2 Biomedical Sciences Training Program, Department of Pathology, University Hospitals Cleveland Medical Center, Case Western Reserve University School of Medicine, Cleveland, OH, USA; 3 Department of Medicine, University of California San Diego, La Jolla, CA, USA; 4 Center for Vaccine Innovation, La Jolla Institute for Immunology, La Jolla, CA, USA; 5 Department of Pathology and Laboratory Medicine, University of Pennsylvania Perelman School of Medicine, Philadelphia, PA, USA; 6 Center for Vaccine Research, Department of Infectious Disease Immunology, Statens Serum Institut, Copenhagen, Denmark

## Abstract

CD4^+^ T cell–mediated control of tuberculosis (TB) requires recognition of macrophages infected with *Mycobacterium tuberculosis* (Mtb). Yet, not all Mtb-specific T cells recognize infected macrophages. Using infected monocyte-derived macrophages and autologous memory CD4^+^ T cells from individuals with stable latent Mtb infection (LTBI), we quantify the frequency of activated T cells. T cell antigen receptor (TCR) sequencing revealed >70% of unique and >90% of total Mtb-specific TCR clonotypes in LTBI are linked to recognition of infected macrophages, while a subset required exogenous antigen exposure, suggesting incomplete recognition. Clonotypes specific for multiple Mtb antigens, and other pathogens, were identified. Remarkably, antigen screening revealed all TCRs to be specific for type VII secretion system (T7SS) substrates. Mtb-specific clonotypes expressed signature effector functions dominated by IFNγ, TNF, IL-2, and GM-CSF or chemokine production and signaling. We propose that TB vaccines, which elicit T cells specific for T7SS substrates, recognize infected macrophages, and express canonical effector functions, will offer protection against TB.

## Teaser

Effective tuberculosis control requires CD4^+^ T cell recognition of infected macrophages. The authors show that TCR clonotypes from latently infected individuals demonstrate dominant but not exclusive recognition of infected macrophages, specificity for type VII secretion system substrates, and expression of canonical effector programs.

## Graphical Abstract

**Figure:**
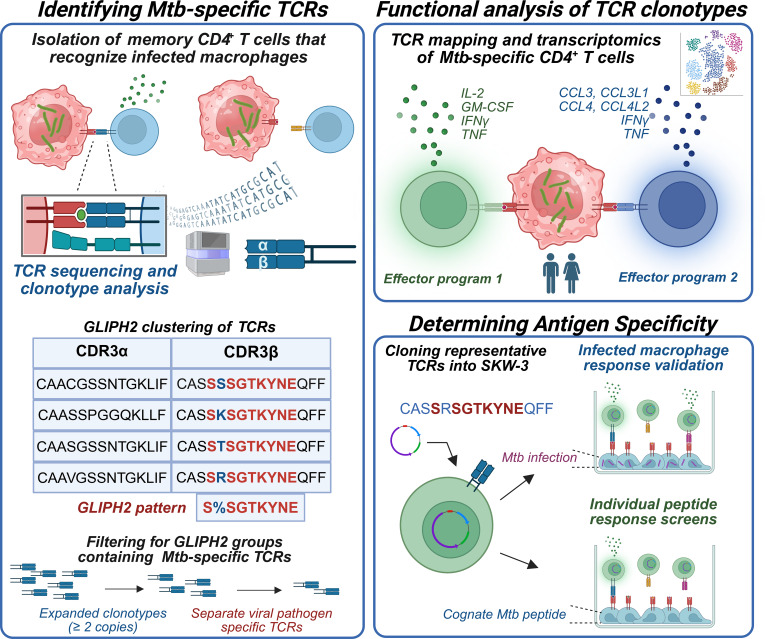

## Introduction

*Mycobacterium tuberculosis* (Mtb), the bacterium that causes tuberculosis (TB), continues to cause more deaths than any other infectious disease (Bagcchi, 2023). There is an urgent need for a vaccine that prevents active TB and reduces Mtb transmission. However, determining the immune cells with the greatest potential for protection through vaccination has proven challenging. CD4^+^ T cells are a major focus of TB vaccine development due to their central and multifaceted role in the immune response to Mtb infection (Bell and Noursadeghi, 2018; Mogues et al., 2001; Bromley et al., 2024). Protection by CD4^+^ T cells was shown to depend on direct recognition of peptide–MHC (pMHC) expressed on infected antigen-presenting cells (APCs) by Mtb-specific T cells (Srivastava and Ernst, 2013). Yet, evidence from the mouse model of TB demonstrates a lack of recognition of infected macrophages by a large fraction of Mtb-specific T cells (Yang et al., 2018; Patankar et al., 2020; Bold et al., 2011). We and others have found that macrophage phenotype (Gail et al., 2023), antigen specificity (Yang et al., 2018), the context of T cell priming (Griffiths et al., 2016; Nyendak et al., 2016; Patankar et al., 2020), TCR avidity and dissociation rate (Gallegos et al., 2016; Nauerth et al., 2013), and mycobacterial virulence factors (Mwebaza et al., 2023) each contribute to T cell recognition of infected cells. For humans, the extent to which CD4^+^ T cells recognize infected macrophages is unexplored.

Upon infection, effector T cells are identified in the lungs only after priming occurs (Wolf et al., 2008; Reiley et al., 2008; Carpenter et al., 2016; Carpenter et al., 2017). T cell priming depends on the transport of live bacteria from the lungs by CCR2^+^ monocyte-derived cells, and on antigen presentation by conventional dendritic cells (DCs) in mediastinal lymph nodes (MLNs) (Samstein et al., 2013; Wolf et al., 2008). Yet, it is macrophages in the lungs that serve as the niche cells for Mtb growth (Russell et al., 2025), making their recognition by T cells critical to protection. Differences in the repertoire of Mtb antigens presented by DCs in lymphoid organs and infected macrophages in the lungs could explain the lack of recognition by a subset of T cells. Using mass spectroscopy, recent studies found only a fraction of the Mtb proteome to be presented by human APCs after infection (Leddy et al., 2023, Leddy et al., 2024, *Preprint*). Since direct measurements of the Mtb peptides presented *in vivo* are impracticable in humans, indirect assessments using TCR sequencing of T cells activated in response to antigen-laden or infected APCs are critical tools.

Individuals who effectively control Mtb infection were found to possess TCRs specific for a range of Mtb antigens (Musvosvi et al., 2023; Huang et al., 2020; Glanville et al., 2017). Conversely, individuals who fail to control infection, and ultimately progress to active TB, exhibit enrichment of alternate TCR motifs (Musvosvi et al., 2023), indicating a difference in their antigen-specific T cell responses. In murine and nonhuman primate models, the effector functions expressed by protective CD4^+^ T cells include canonical IFNγ, TNF, and IL-2 (Lewinsohn et al., 2017), GM-CSF (Rothchild et al., 2017; Dis et al., 2022), cytotoxicity (Gideon et al., 2022), and, depending on the context, IL-17 or IL-10 production (Dijkman et al., 2019; Bromley et al., 2024). In humans, latent Mtb infection (LTBI) that is “stable” (i.e., did not progress to active TB) has been associated with protection (Andrews et al., 2012). Increased memory CD4^+^ T cell responses were observed among individuals vaccinated with the M72/AS01_E_ (Rodo et al., 2019), a candidate TB vaccine linked to ∼50% reduction in TB in a Phase 2B clinical trial (Tait et al., 2019). Despite promising efficacy with M72/AS01_E_, a major challenge in TB vaccine development is the lack of a reference for protective T cell function. In contrast, other TB vaccine candidates have not yet shown protection (Tameris et al., 2013; Darrah et al., 2014; Nemes et al., 2018). Human T cell clones derived from recipients of the AERAS-402 vaccine lacked recognition for Mtb-infected cells despite responding to peptide-laden APCs (Nyendak et al., 2016). These findings suggest the ability to recognize infected macrophages could serve as a marker of protective T cells if linked to clinical protection from TB.

In this study, we sought to quantify the proportion of peripheral blood human memory CD4^+^ T cells that recognize infected macrophages. Using *ex vivo* coculture of primary human CD4^+^ T cells with autologous Mtb-infected macrophages, we tested the hypothesis that a subset of memory CD4^+^ T cells from individuals with LTBI lack efficient recognition. Among 10 individuals with stable LTBI, the majority of Mtb-specific CD4^+^ T cells became activated in response to infected macrophages, whereas a subset required exogenous antigen exposure, suggesting incomplete recognition. We used single-cell TCR sequencing (scTCRseq) to refine our estimates of CD4^+^ T cell recognition of infected macrophages and were surprised to identify viral pathogen-specific TCR clonotypes, suggesting bystander activation. Strikingly, by clustering TCRs using Grouping of Lymphocyte Interactions by Paratope Hotspots V2 (GLIPH2), several of our top GLIPH2 groups were equivalent to those recently published, containing TCRs annotated as specific for the CFP10, EspA, and mIHF antigens (Glanville et al., 2017; Musvosvi et al., 2018; Huang et al., 2020). Antigen screening of additional GLIPH2 groups responsive to infected macrophages revealed TCRs specific for the secreted T7SS substrates PE12, PE18, PE19, EsxG, EsxS, EspC, and CFP10 from Mtb. Using single-cell transcriptomics (scRNAseq), we distinguished the effector functions of memory CD4^+^ T cells enriched with either Mtb-specific or viral antigen–specific TCRs. We propose that the identification of TCRs, antigen specificities, and effector functions linked to CD4^+^ T cell recognition of infected macrophages will inform TB vaccine design.

## Results

### A subset of memory CD4^+^ T cells lack recognition for Mtb-infected macrophages

**Figure 1.**
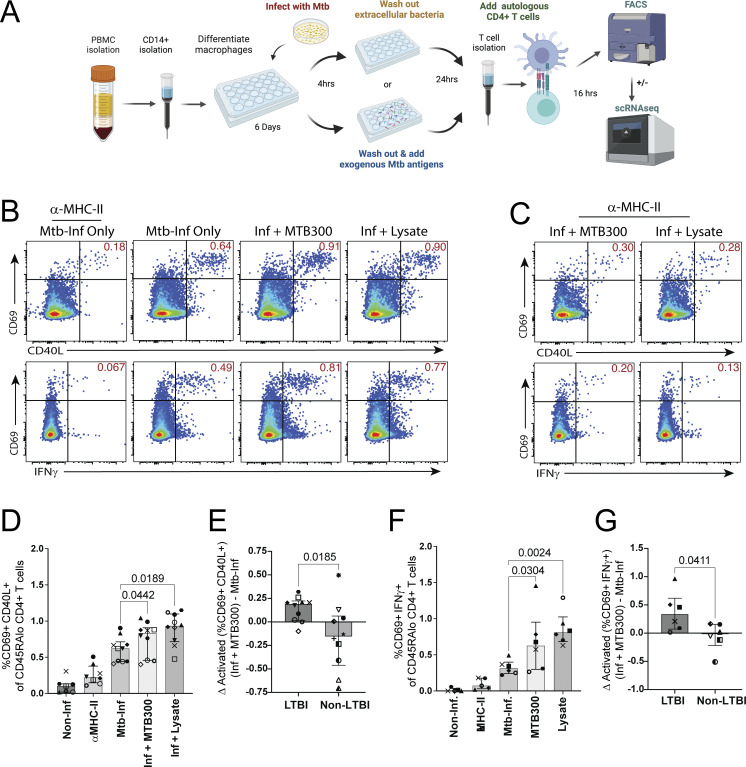
**Subset of memory CD4** ^**+**^ **T cells lack recognition for Mtb-infected macrophages. (A)** Schematic of experimental workflow to coculture infected macrophages with autologous memory CD4^+^ T cells for flow cytometry or sorting. Created in BioRender. Carpenter, S. (2025) https://BioRender.com/v53j172. **(B and C)** Flow cytometry plots from a representative experiment comparing activation marker co-expression of CD69 with CD40L (top row) or IFNγ (bottom row), (B) gated on CD45RA^Lo^ CD4^+^ T cells after 16–18 h coculture with Mtb-infected macrophages ± treatment with MTB300 or lysate, and (C) in the presence of α-MHC-II blocking antibodies. Data are representative of 10 (CD69 vs. CD40L) and 6 (CD69 vs. IFNγ) experiments and participants. **(D and E)** Summary bar graphs compare (D) median (and IQR) co-expression of CD69 and CD40L, and (E) the difference in activation when MTB300 is added to infected macrophages (10 LTBI and 7 non-LTBI participants). **(F and G)** Summary bar graphs compare (F) median (and IQR) CD69 and IFNγ co-expression, and (G) change in activation when MTB300 is added (6 LTBI and 6 non-LTBI participants). Each symbol represents the mean of one to three replicates from independent experiments. Statistical significance was determined by the Wilcoxon matched-pairs signed rank test.

**Figure S1.**
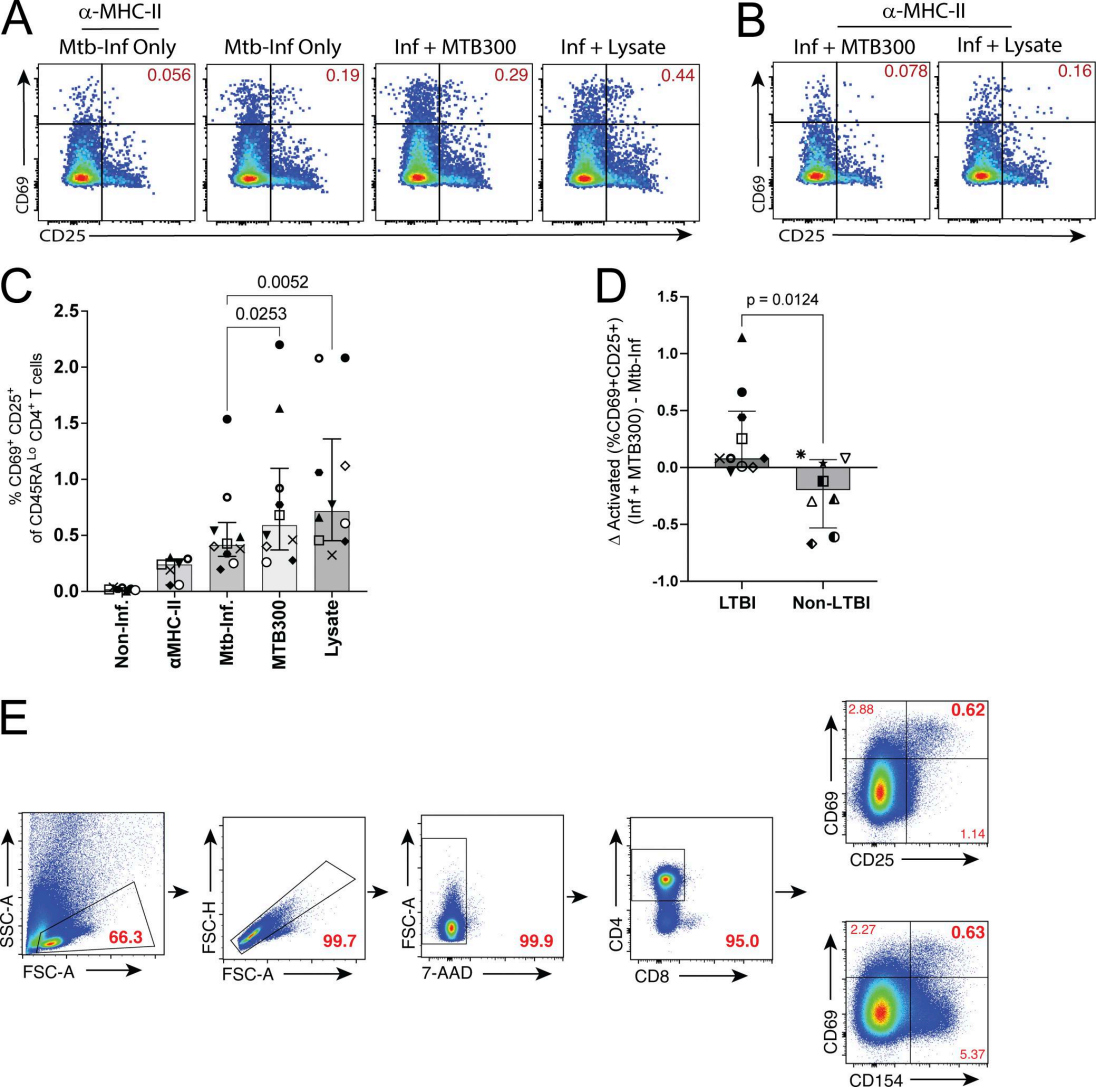
**CD69 and CD25 co-expression reveals a subset of memory CD4** ^**+**^ **T cells that lack recognition of Mtb-infected macrophages.** Related to Figs. 1 and 2. **(A and B)** (A) Flow cytometry plots comparing the co-expression of CD69 and CD25, gated on CD45RA^Lo^ CD4^+^ T cells after 16–18 h coculture with Mtb-infected (MOI 4–5) macrophages either alone or with addition of exogenous antigens (MTB300 or lysate) and (B) with and without α-MHC-II blocking antibodies. Plots are concatenated from three replicates from one experiment; data are representative of 10 independent experiments each with two to three replicates per condition. **(C and D)** (C) Summary bar graphs compare mean (± SEM) co-expression of CD69 and CD25 (10 participants), and (D) the difference in activation when MTB300 is added to Mtb-infected macrophages for samples from 10 LTBI and 6 non-LTBI participants. **(E)** Representative flow plots showing the gating of memory CD4^+^ T cells activated in response to Mtb-infected macrophages (or controls). After gating on lymphocytes (SSC Area [SSC-A] vs. FSC Area [FSC-A]) and single cells (FSC Height (FSC-H] vs. FSC-A), live CD4^+^ 7-AAD^Lo^ T cells were identified. Co-expression of CD69 and either CD25 or CD40L was used to identify activated T cells for sorting.

To determine the proportion that recognize Mtb-infected macrophages, we isolated memory (CD45RA^Lo^) CD4^+^ T cells and CD14^+^ monocytes from the peripheral blood of healthy individuals with stable LTBI. We enrolled 10 healthy adult volunteers with a history of exposure to TB and a positive IFNγ release assay (IGRA) or tuberculin skin test (TST) >10 mm obtained at least 3 years prior to participating (see Materials and methods). After monocyte-derived macrophage (MDM) differentiation and infection with Mtb strain H37Rv at an MOI of 4–5, we cocultured infected MDMs with autologous memory CD4^+^ T cells (Fig. 1 A). We previously found that infection with H37Rv at an MOI of 4-5 leads to infection of >75% and 95% of M1- and M2-differentiated human macrophages, respectively (Gail et al., 2023). 16–18 h later, activation-induced markers (AIMs) and IFNγ secretion were used to quantify the subset of memory CD4^+^ T cells activated in response to Mtb-infected macrophages, as performed previously (Gail et al., 2023, 2024; Reiss et al., 2017; Huang et al., 2020; Musvosvi et al., 2023). To identify Mtb-specific memory CD4^+^ T cells lacking efficient recognition, we compared the proportion of T cells activated in response to infected macrophages vs. infected macrophages to which MTB300 megapool peptides (Lindestam Arlehamn et al., 2016) or H37Rv whole-cell lysate (lysate) was added (Fig. 1 A). MTB300 or lysate was added to Mtb-infected rather than noninfected MDMs to control for the effects of macrophage activation and inflammatory cytokines on T cell activation. Consistent with our hypothesis, the greater numbers of memory CD4^+^ T cells co-expressed the AIMs CD69 and CD40L in response to infected macrophages treated with MTB300 (0.833% [0.458–0.908]; median [interquartile range (IQR)], 10 participants) or lysate (0.933% [0.715–1.098]) compared with infection-only (0.62% [0.443–0.713]) (Fig. 1, B–E). Interestingly, most but not all participants’ CD4^+^ T cell responses increased with the addition of MTB300 (8 of 10 individuals) or lysate (9 of 10 individuals), suggesting response heterogeneity among individuals (Fig. 1, D and E). The same trend was observed for IFNγ^+^ secretion by CD69^+^ CD4^+^ T cells in response to infected macrophages (0.312% [0.243–0.398], median [IQR], 6 participants) and + MTB300 (0.626% [0.299–0.952]) or + lysate treatment (0.808% [0.685–1.03]) (Fig. 1, B and F), and for CD69 and CD25 co-expression (Fig. S1, A–C). The reduction in T cell activation with α-MHC-II (HLA-DR/-DQ/-DP) mAb blockade indicates TCR-mediated activation (Fig. 1, B–D; and Fig. S1, A–C). The increased proportion of memory CD4^+^ T cells activated when MTB300 was added preferentially occurred among participants with LTBI, while little change occurred for healthy control participants without LTBI (non-LTBI) (Fig. 1, E and G; and Fig. S1 D). Therefore, the increase in activated T cells observed when exogenous Mtb antigens were added suggested dominant but incomplete recognition of infected macrophages by Mtb-specific memory CD4^+^ T cells.

### TCR sequencing identifies clonotypes linked to recognition of infected macrophages

**Figure 2.**
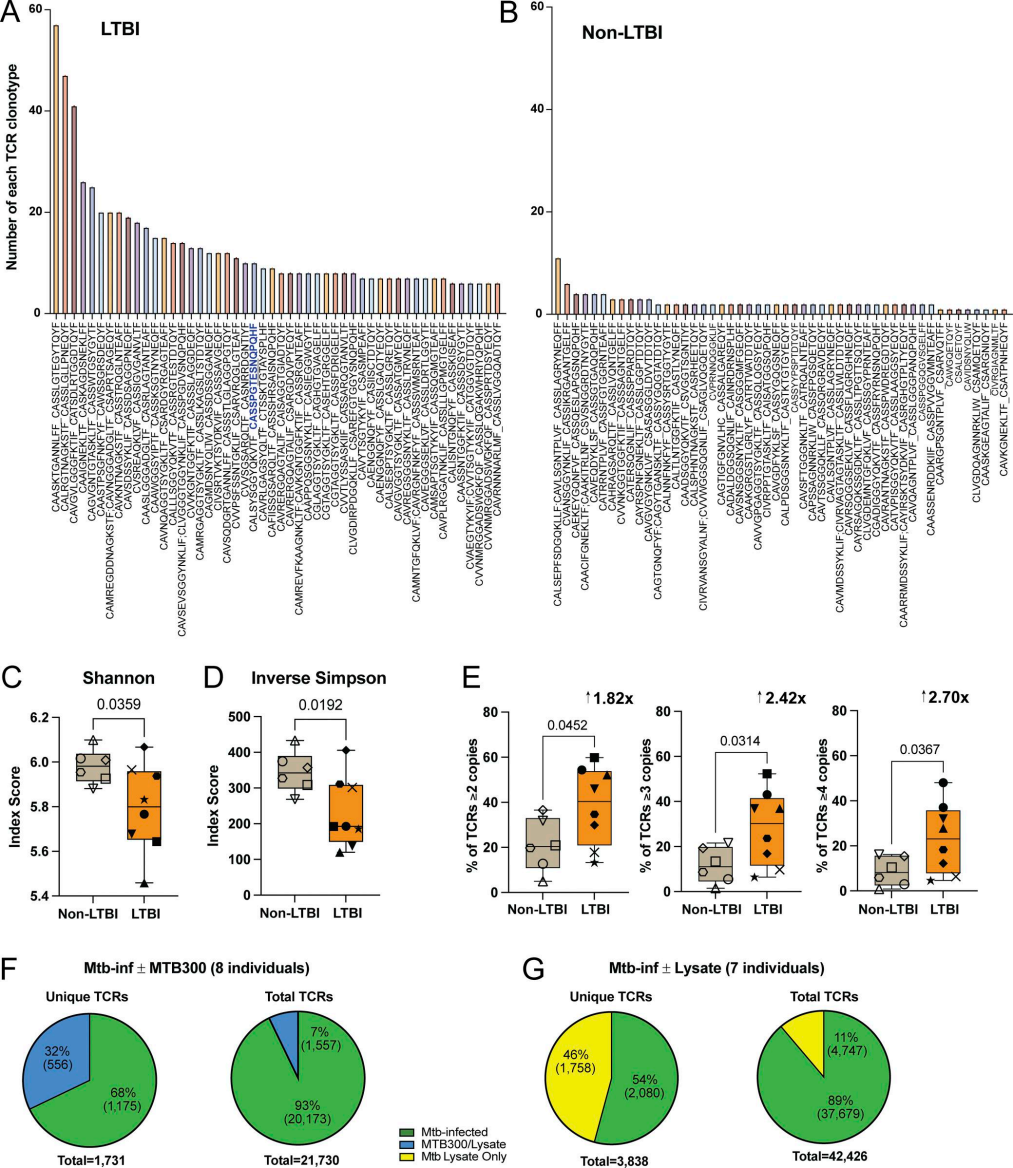
**TCR sequencing identifies clonotypes linked to recognition of infected macrophages. (A and B)** Bar graphs of the top 50 TCR clonotypes and their frequencies from representatives of (A) eight LTBI and (B) six non-LTBI participants. TCR clonotypes are displayed as CDR3α_CDR3β; some TCRs contained two CDR3α or CDR3β chains. Blue font highlights a CDR3β motif previously annotated as specific for EspA_301-315_. **(C and D)** Summary box plots of median (with IQR and range) (C) Shannon and (D) Inverse Simpson index scores for eight LTBI and six non-LTBI participants. Each symbol represents individual participant TCR repertoires. **(E)** Summary box plots of median (with IQR and range) percentage of TCR clonotypes present in ≥2, 3, or 4 copies with fold differences listed above each graph. Statistical significance was determined by unpaired Welch’s *t* test. **(F and G)** Pie charts showing percentage (and number) of unique (left) and total (right) TCRβ sequences linked to (F) responses to infected macrophages (green) or after adding MTB300 (blue), and (G) responses to infected macrophages (green) or after adding lysate (yellow), combined from seven to eight individuals.

AIMs are most useful in the context of T cell stimulations with peptides. However, Mtb infection (and lysate) can elicit “bystander” T cell activation, presumably through inflammatory cytokines, leading to an overestimation of the Mtb-specific response (Reiss et al., 2017; Gail et al., 2023). Therefore, we used scTCRseq to refine our estimates of CD4^+^ T cell recognition of infected macrophages. Following coculture with infected MDMs (± MTB300 or lysate), AIM^+^ memory CD4^+^ T cells were flow-sorted and high-throughput single-cell sequencing was performed (Fig. 1 A and Fig. S1 E). Among the top 50 CDR3β sequences from representative LTBI and non-LTBI participants, clonally expanded TCRs were frequently identified in LTBI (Fig. 2, A and B). Greater TCR clonality was observed among individuals with LTBI, compared with non-LTBI controls as indicated by lower Shannon and Inverse Simpson indices (Fig. 2, C and D). Among individuals with LTBI, a mean of ∼40% of unique TCRs were clonally expanded, compared with ∼20% for non-LTBI (Fig. 2 E, left panel). Upon examining TCRs present in ≥3 or ≥4 copies, more clonotypes were identified for LTBI vs. non-LTBI participants (Fig. 2 E, middle and right panels). Since 16–18 h is not sufficient to elicit T cell proliferation *in vitro*, clonal T cell expansions were determined to have previously occurred *in vivo* in response to infection. Interestingly, among the top 50 TCRs, the CDR3β sequence “CASSPGTESNQPQHF” was identified in LTBI (Fig. 2 A), a TCR previously observed in a South African LTBI cohort and found to have specificity for the Mtb antigen EspA_301-315_ (Huang et al., 2020).

We next sought to create a condition where a set of infected macrophages also received exogenous treatment with MTB300 peptides, followed by coculture with autologous memory CD4^+^ T cells. This enabled us to compare the TCR repertoires among responses to Mtb-infected macrophages ± MTB300 peptides. We found the majority of unique and total expanded TCR clonotypes were linked to recognition of infected macrophages, but a subset of 7–32% required exposure to MTB300 (Fig. 2 F). Like MTB300, new clonotypes were also identified when lysate was added to infected macrophages (Fig. 2 G). Yet, the majority of both unique and total TCRs were again linked to recognition of infected macrophages. Therefore, scTCRseq, and a focus on expanded TCR clonotypes, revealed dominant but incomplete recognition of infected macrophages by memory CD4^+^ T cells.

### GLIPH2 refines estimates of Mtb-specific CD4^+^ T cell recognition of infected macrophages

**Figure 3.**
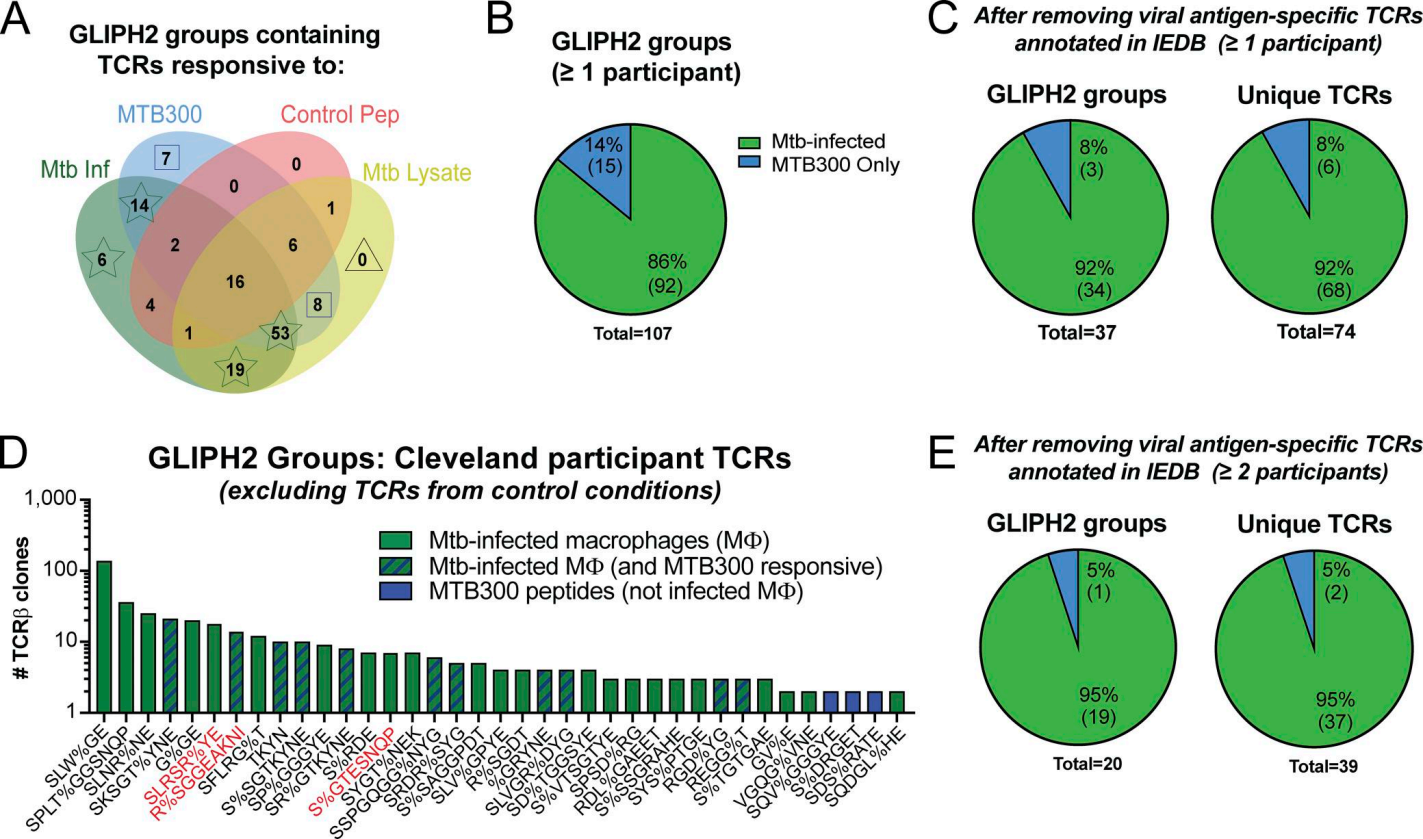
**GLIPH2 refines estimates of Mtb-specific CD4** ^**+**^ **T cell recognition of infected macrophages. (A)** Venn diagram indicating the number of GLIPH2 groups containing TCRs linked to responses to infected macrophages (green, stars), MTB300 (added to infected macrophages or to PBMCs) (blue, squares), control peptide megapools added to PBMCs (red), or lysate (added to infected macrophages) (yellow, triangle). **(B)** Pie charts of percentage (and number) of remaining GLIPH2 groups that respond to infected macrophages (green; sum of star groups from A) or MTB300 only (blue; sum of square groups from A). **(C)** Pie charts of GLIPH2 groups (left) and corresponding unique TCRβs (right) after removing GLIPH2 groups containing TCRs linked to viral antigen responses. **(D)** Bar graph of GLIPH2 groups (x axis) estimated to be Mtb-specific, rank-ordered by the sum of the highest number of TCR copies per experimental condition (y axis). Responses to infected macrophages (green), MTB300 only (blue), or both (blue stripes) are indicated. “%” indicates any amino acid substitution. **(E)** Pie charts comparing responses to infected macrophages (green) or MTB300 peptides only (blue) for GLIPH2 groups (left) containing unique TCRβs (right) contributed by ≥2 participants. Data were generated from a combined list of expanded TCRs from all experimental conditions (10 experiments; 10 LTBI and 6 non-LTBI participants).

**Figure S2.**
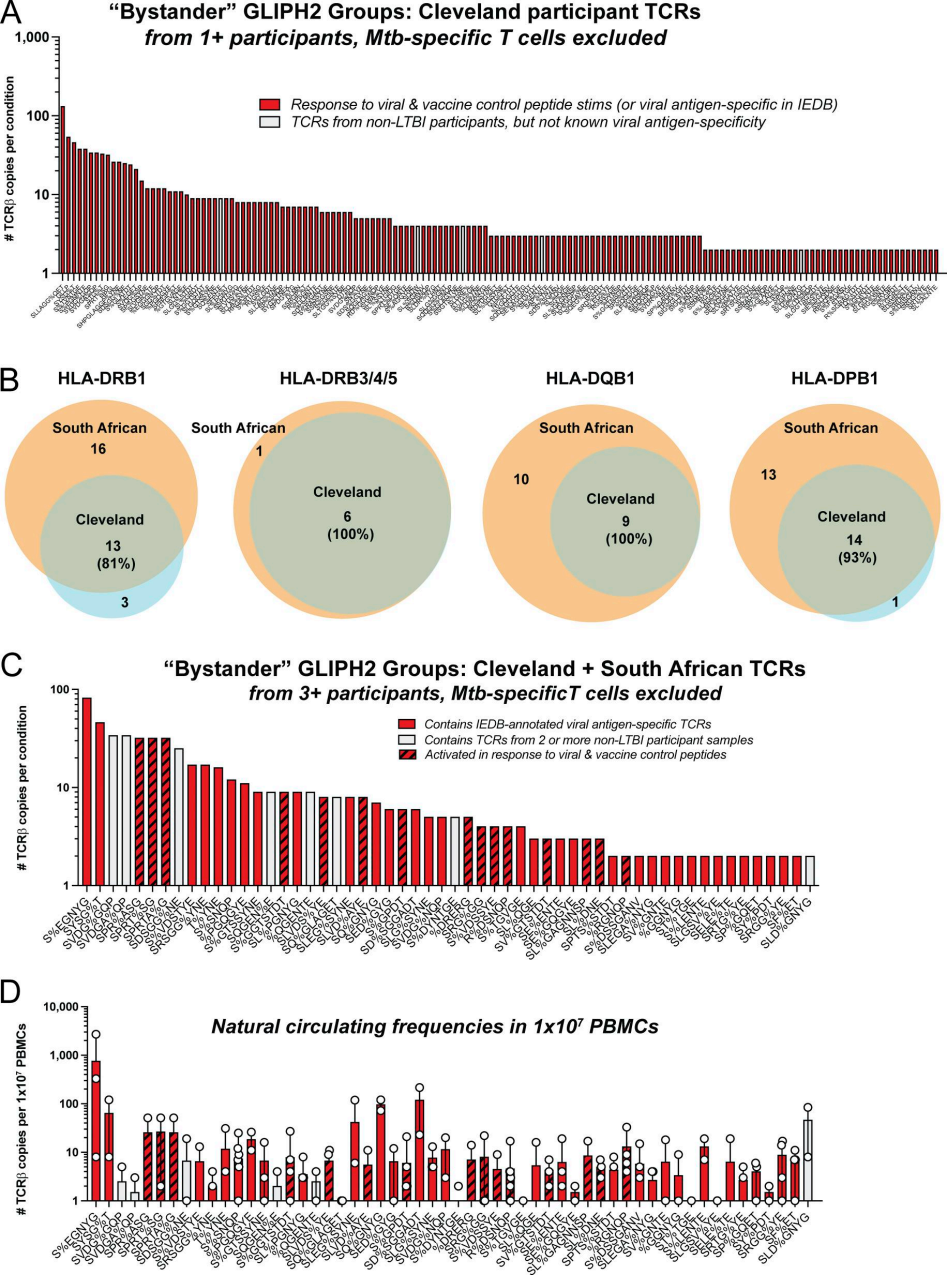
**Distribution of GLIPH2 groups linked to viral or vaccine control responses.** Related to Figs. 3 and 4. **(A)** Bar graph of GLIPH2 groups (x axis) from ≥1 Cleveland participant estimated to be viral or vaccine antigen–specific. **(B)** Venn diagrams of numbers of HLA-II alleles shared (or not) between Cleveland and South African participants and percentage of Cleveland HLA alleles expressed by South African participants. **(C)** Bar graph of GLIPH2 groups (x axis) from ≥3 participants from the combined list of Cleveland and South African TCRs estimated to be viral or vaccine antigen–specific, based on IEDB annotation (red), non-LTBI participants (gray), or control peptide stimulations (stripes), rank-ordered by the sum of the highest TCR copy number (y axis) per condition. **(D)** Bar graph of mean circulating frequency of Cleveland participants’ TCRβ clonotypes (symbols) as constituents of each GLIPH2 group from the combined list of TCRs after cross-referencing unstimulated PBMCs from Cleveland participants.

We next took several steps to focus our analysis on clonotypes that are Mtb-specific. First, we stimulated whole peripheral blood mononuclear cells (PBMCs) from the same individuals with either MTB300 or a combination of “control” viral and vaccine peptide megapools from cytomegalovirus (CMV), Epstein-Barr virus (EBV), *Bordetella pertussis*, tetanus toxoid (Dan et al., 2016; da Silva Antunes et al., 2017; Carrasco Pro et al., 2015), and the SARS-CoV-2 spike protein. After 16–18 h, we flow-sorted the AIM^+^ CD4^+^ T cells and performed scTCRseq, identifying TCR clonotypes that responded either to MTB300 or to control peptides. Following peptide stimulations, we cross-referenced peptide-elicited TCR clonotypes with those found among responses to infected macrophages and separated clonotypes activated in response to control peptides (Fig. 3 A). Second, we used GLIPH2 (Huang et al., 2020; Musvosvi et al., 2023) to group the expanded TCRs (≥2 copies) from a list of TCR sequences combined from all experimental conditions, including responses to infected macrophages ± MTB300, infected macrophages ± lysate, and peptide stimulations using either control or MTB300 peptide pools. After removing GLIPH2 groups that contained TCRs linked to control peptide responses (Fig. 3 A), we identified 107 groups that met statistical criteria for CDR3 motif enrichment, Vβ gene usage, and CDR3 length (see Materials and methods). Of these, 92 GLIPH2 groups (86%, ☆) were linked to recognition of Mtb-infected macrophages, while 15 (14%, ⊡) responded to MTB300 peptides but not infected macrophages (Fig. 3, A and B). Third, we searched for these TCRβ sequences in the Immune Epitope Database (IEDB, https://www.iedb.org) (Chronister et al., 2021) and removed GLIPH2 groups that contained TCRs with ≥97% CDR3β homology with TCRs annotated as specific for viral antigens (Fig. S2 A). Finally, we eliminated five additional GLIPH2 groups that contained TCRs from non-LTBI participant responses to infected macrophages (Fig. S2 A).

Of the remaining 37 GLIPH2 groups and 74 unique TCR clonotypes, 92% were linked to recognition of infected macrophages and 8% to MTB300 peptides only (Fig. 3, C and D). Three of these motifs (SLRSR%YE, R%SGEAKNI, and S%GTESNQP) were homologous or identical to those published in three recent studies of LTBI in South Africa, containing TCRs specific for the Mtb antigens mIHF (Rv1388), CFP10 (Rv3874, EsxB), and EspA (Rv3616c), respectively (Musvosvi et al., 2023; Huang et al., 2020; Glanville et al., 2017). Their discovery among our LTBI participants indicated our approach enriched for Mtb-specific TCRs. Finally, GLIPH2 groups containing TCRs found among ≥2 participants showed a similar trend where the majority (95%) were linked to recognition of infected macrophages (Fig. 3 E).

### TCRs from additional LTBI cohorts enhance the ability of GLIPH2 to distinguish Mtb-specific clonotypes

**Figure 4.**
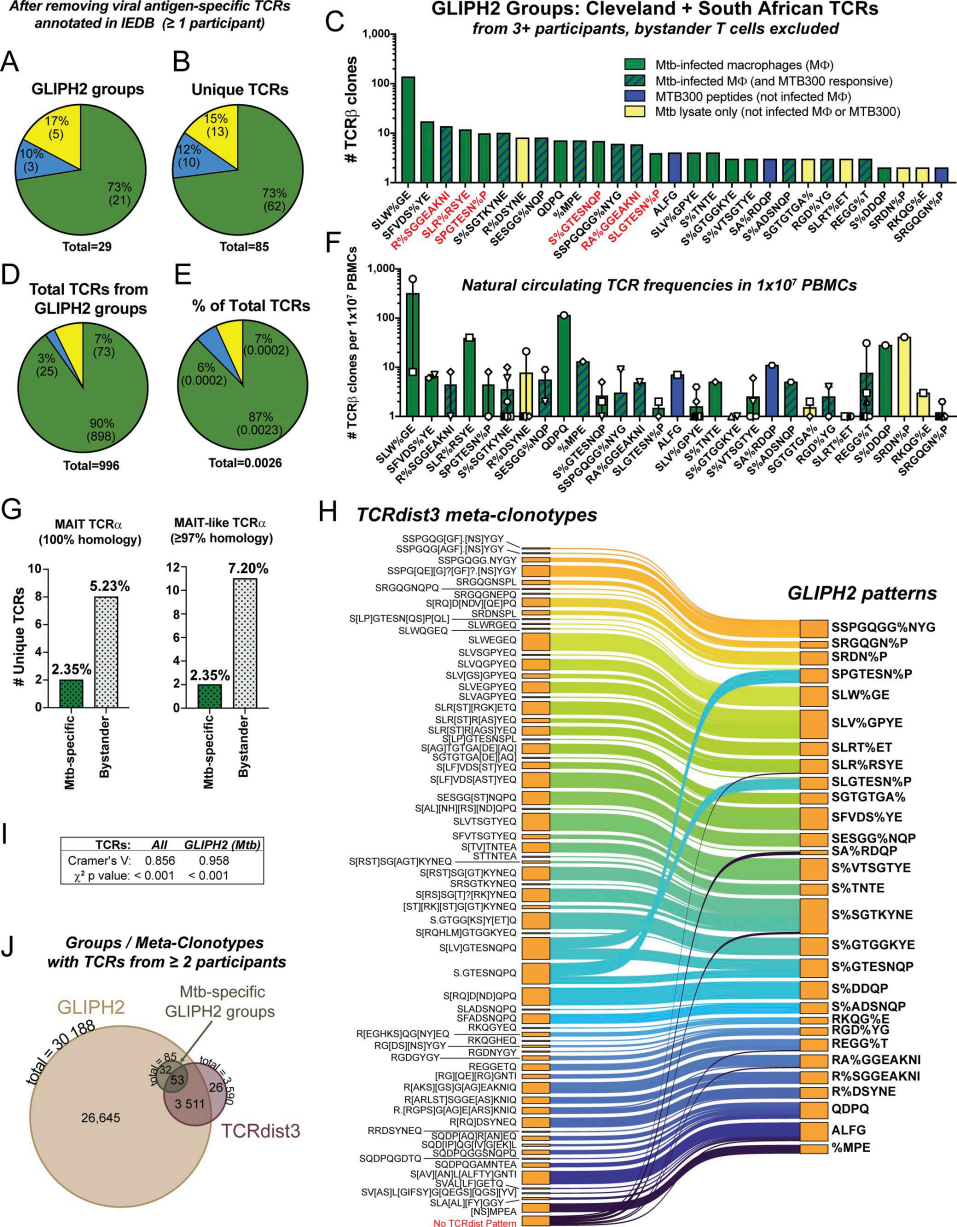
**TCRs from additional LTBI cohorts enhance the ability of GLIPH2 to distinguish Mtb-specific clonotypes. (A and B)** Pie charts comparing percentage (and number) of (A) GLIPH2 groups or (B) unique TCRβs linked to a response to Mtb-infected macrophages (green), MTB300 only (blue), or lysate only (yellow). **(C)** Bar graph of GLIPH2 groups estimated to be Mtb-specific (x axis) containing TCRs contributed by ≥3 participants, rank-ordered by the sum of the highest number of TCR copies recovered in an experiment (y axis). Responses to infected macrophages (green), MTB300 only (blue), both (blue stripes), or lysate only (yellow) are indicated. GLIPH2 groups containing TCRs previously annotated as Mtb antigen–specific in IEDB are in red font; % indicates any amino acid substitution. **(D and E)** Pie charts comparing (D) total or (E) percentage of total CD4^+^ T cell TCRβs sequenced from 10 × 10^6^ unstimulated PBMCs after cross-referencing GLIPH2 groups from Cleveland participants’ responses to infected macrophages (± MTB300 or lysate) to determine natural circulating frequency. **(F)** Bar graph of the mean number of each TCRβ clonotypes (different symbols) sequenced from unstimulated PBMCs (for Cleveland participants only), corresponding to each GLIPH2 group. **(G)** Bar graphs of the number (and percentage) of unique TCRs within GLIPH2 groups estimated to be Mtb-specific or non–Mtb-specific bystander GLIPH2 groups that contain either 100% (left) or 97–99% (right) CDR3α homology with MAIT cells. **(H)** Comparison of TCRdist3 meta-clonotypes generated from all TCRs in respective GLIPH2 groups estimated to be Mtb-specific in C. Thickness of each line corresponds to the number of TCR sequences across all samples. For meta-clonotypes, “.” indicates any amino acid substitution (analogous to % in GLIPH2), “?” indicates an optional amino acid, and brackets indicate an either–or substitution. **(I)** Chi-square statistics comparing membership for all TCRs (*All*) or only the GLIPH2 groups in C (*GLIPH2* [*Mtb*]). **(J)** Proportional Venn diagram showing the number of unique TCRs clustered by GLIPH2 or TCRdist3 within groups (or meta-clonotypes) with TCRs from ≥2 participants. The green circle represents TCRs in GLIPH2 groups estimated to be Mtb-specific from C. TCRs grouped by GLIPH2 and TCRdist3 were compared using a chi-square analysis and Cramer’s V post hoc test.

Our sample size of 16 participants (10 LTBI, 6 non-LTBI) limited our ability to ensure GLIPH2 groups were robust by using HLA associations or containing TCRs from >2 participants (Musvosvi et al., 2023). To enrich our dataset, we added TCRs from 106 participants in three recent publications of TCRs for CD4^+^ T cells activated in response to Mtb antigens in the form of Mtb lysate, MTB300 peptides, or overlapping peptide libraries spanning the ESAT6 and CFP10 antigens (Glanville et al., 2017; Huang et al., 2020; Musvosvi et al., 2022; Musvosvi et al., 2023). Representation of HLA alleles from Cleveland participants in the South African cohorts was 81–100% (Fig. S2 B). Using the combined dataset, we focused only on GLIPH2 groups that contained TCRs from ≥3 individuals with a significant HLA association score, as described previously (Musvosvi et al., 2023), in addition to our previous criteria, and containing TCRs from Cleveland LTBI participants. 56 GLIPH2 groups were categorized as not specific for Mtb antigens (Fig. S2 C). We identified 29 GLIPH2 groups with 85 unique TCRs from ≥3 participants that did not contain TCRs from control peptide stimulations, non-LTBI participants, or viral antigen–specific TCRs annotated in the IEDB (Fig. 4, A and B; and Data S1). 73% of GLIPH2 groups (and 73% of TCR clonotypes) estimated to be Mtb-specific were linked to recognition of infected macrophages (Fig. 4, A and B). 12% of TCRs within GLIPH2 groups responded to MTB300 but not infected macrophages, and 15% to lysate but not MTB300 or infected macrophages (Fig. 4 B). GLIPH2 groups linked to recognition of infected macrophages contained more TCR copies per sample (Fig. 4 C). Many were the same as GLIPH2 groups identified in the Cleveland participants’ TCRs (Fig. 3 D). In the combined analysis, eight of these (SLRSR%YE, R%SGGEAKNI, and RA%GGEAKNI—“GEAK” motifs; SPGTESN%P, SLGTESN%P, and S%GTESNQP—“TESN” motifs; and SRDN%P and ALFG) were the same as those previously published (Musvosvi et al., 2023; Glanville et al., 2017; Huang et al., 2020) revealing specificity for mIHF, CFP10, EspA, and two Mtb antigens yet to be defined, respectively (Fig. 4 C).

To estimate the natural circulating frequencies of each TCR from Cleveland participants within these GLIPH2 groups, we performed TCRβ deep sequencing of bulk peripheral blood CD4^+^ T cells from 10 × 10^6^ PBMCs for each donor. We then cross-referenced the CDR3β sequences from each of the 29 GLIPH2 groups with TCRs sequenced from unstimulated PBMCs. Overall, 90% of the total TCRs enumerated from unstimulated CD4^+^ T cells were linked to clonotypes that recognized infected macrophages, representing a circulating frequency of 0.0023% (Fig. 4, D and E). Several of the GLIPH2 groups containing TCRs that responded only to MTB300 peptides (ALFG, SA%RDQP) or lysate (R%DSYNE, SRDN%P) had comparable frequencies in peripheral blood to TCRs linked to recognition of infected macrophages, suggesting they represent clinically meaningful clonotypes (Fig. 4 F). Some bystander TCRs, responsive to viral or vaccine antigens, were at least as frequent in circulation as the Mtb-specific clonotypes (Fig. S2 D). In summary, most GLIPH2 groups estimated to contain Mtb-specific TCRs were linked to recognition of infected macrophages and outnumbered those that only responded to MTB300 or lysate ∼9:1 in circulation in stable LTBI.

Next, we compared canonical CDR3α sequences of CD1d-restricted invariant natural killer T (iNKT), CD1b-restricted germline-encoded mycolyl lipid–reactive (GEM) T, and mucosal-associated invariant T (MAIT) cells with TCRs from all GLIPH2 groups. While no iNKT or GEM T cell homology was identified, two Mtb-specific TCRs (from SFVDS%YE) and eight from bystander GLIPH2 groups contained MAIT cell CDR3α sequences (Fig. 4 G; and Datas S1 and S2). Furthermore, two Mtb-specific and 11 bystander clonotypes contained MAIT-like CDR3α homology. These data suggest MAIT cells were more enriched in bystander GLIPH2 groups.

Finally, to evaluate TCR clustering with another algorithm, we used TCRdist3 (Mayer-Blackwell et al., 2021). TCRdist3 generated more meta-clonotypes than GLIPH2 groups (67 vs. 29); each meta-clonotype contained fewer TCRs on average (Fig. 4 H). Nearly all TCRs from the 29 Mtb-specific GLIPH2 groups were also clustered by TCRdist3, and strong concordance between TCRs grouped by either algorithm was observed (Fig. 4, H and I). Using TCRdist3, 3,590 TCRs were clustered into meta-clonotypes that contained TCRs from at least two individuals, and 3,564 were also grouped by GLIPH2 (Fig. 4 J). Among the 85 unique TCRs in 29 GLIPH2 groups, 53 (62%) were also found in meta-clonotypes containing TCRs from ≥2 participants, while 32 were constituents of meta-clonotypes containing TCRs from a single participant or were not clustered (Fig. 4 J and Data S3). Six of the 32 TCRs are from GLIPH2 groups with confirmed specificity for Mtb antigens and would not have been identified using TCRdist3 meta-clonotypes with a threshold of ≥2 participants. Thus, while TCRs grouped by either algorithm were highly overlapping, their distributions into broad (GLIPH2) or narrow (TCRdist3) groups differed.

### TCRs from GLIPH2 groups are specific for Mtb antigens

**Figure 5.**
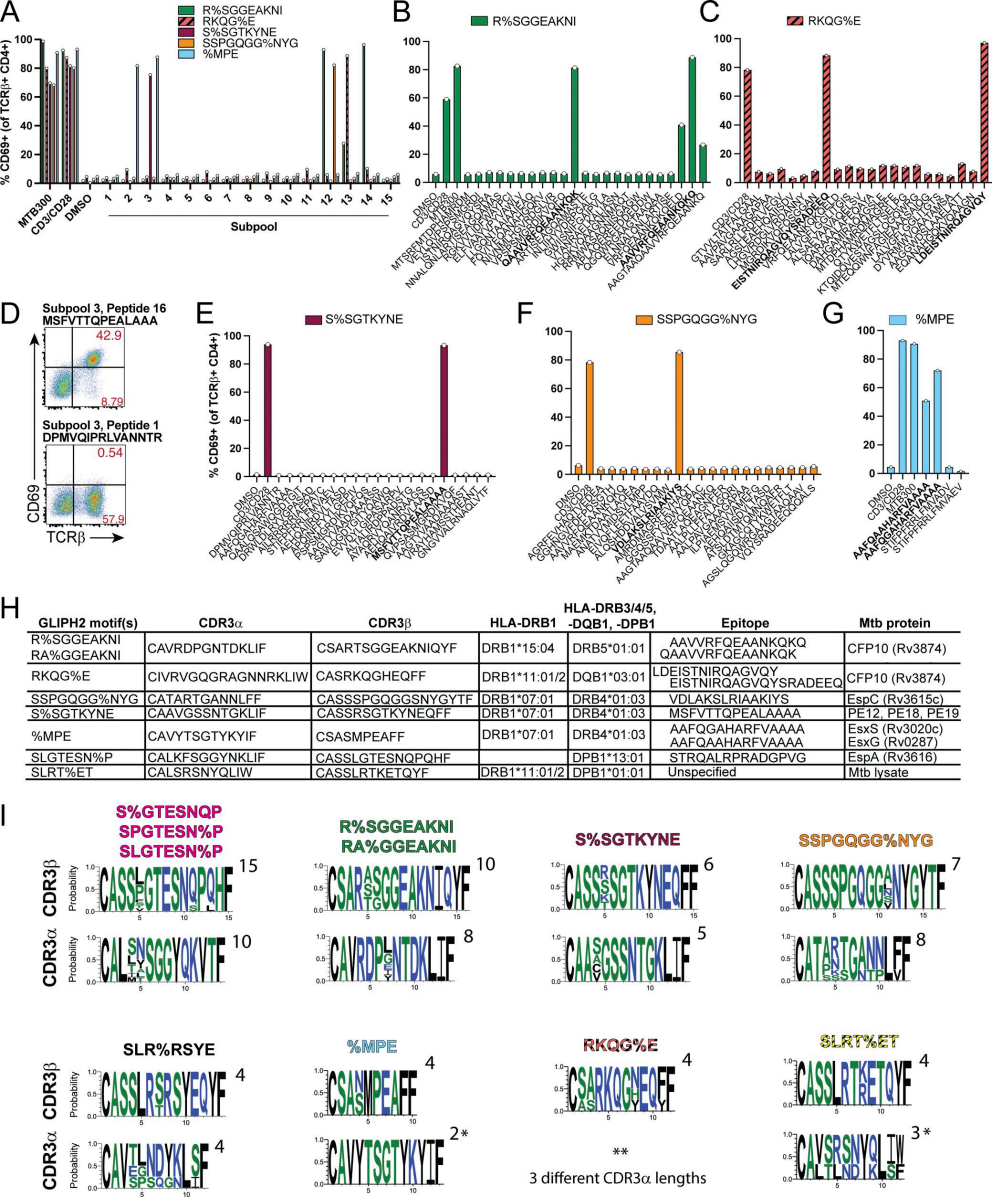
**TCRs from GLIPH2 groups are specific for Mtb antigens. (A–C)** (A) Bar graphs of CD69 expression by flow cytometry (gated on CD4^+^ TCRβ^+^ Live-Dead^Lo^) for SKW-3 cells transduced with a TCR from indicated GLIPH2 groups 18 h after coculture with autologous B cells loaded with MTB300 megapool, 20-peptide subpools, or (B) with individual peptides, DMSO, or after treatment with αCD3/CD28 mAb-coated beads for R%SGGEAKNI and (C) RKQG%E TCR-transduced SKW-3 cells. **(D)** Representative flow plots of CD69 and TCRβ expression gated on total CD4^+^ SKW-3 cells after transduction with a TCR expressing the S%SGTKYNE motif in response to cognate peptide (top) or irrelevant peptide (bottom). **(E–G)** Bar graphs of CD69 expression (of CD4^+^ TCRβ^+^) for (E) the S%SGTKYNE TCR, (F) the SSPGQGG%NYG TCR, and (G) the %MPE TCR in response to individual peptides. **(H)** Single values for each condition are plotted, representing two to three independent experiments for (H) each cloned TCR and respective GLIPH2 group. Cognate peptides and GLIPH2-predicted HLA restrictions are listed. **(I)** Sequence logo plots show the probability of each amino acid for CDR3β (top) and CDR3α (bottom) motifs for each GLIPH2 group containing TCRs where Mtb antigen specificity was established. Created using WebLogo3. Numbers of CDR3 sequences used for each plot are indicated (top-right). * indicates two, and ** indicates three different CDR3α lengths and a lack of CDR3α consensus within the GLIPH2 group.

To determine the antigen specificities of clonotypes linked to recognition of infected macrophages, we cloned representative TCRs from GLIPH2 groups whose expected cognate peptides were contained within MTB300. We cloned each TCR using lentiviral transduction (Dezfulian et al., 2023) (Data S4) into the SKW-3 lymphocytic leukemia cell line, which lacks TCR expression (Shima et al., 1986). We performed antigen screening using autologous B cells loaded with the MTB300 megapool, 20-peptide “subpools,” and individual peptides within MTB300 (Data S5), and identified cognate peptides for five TCRs. At least one subpool led to CD69 expression for each TCR-transduced cell line, indicating specificity for peptides within MTB300 (Fig. 5 A). The CFP10 peptide “AAVVRFQEAANKQKQ” was reported as the target of a GEAK motif TCR (Musvosvi et al., 2023; Glanville et al., 2017) and was recently shown to be presented by human monocyte-derived DCs (MDCs) upon infection with Mtb (Leddy et al., 2024, *Preprint*). Peptides from subpools 12, 13, and 14 containing this sequence led to CD69 expression by SKW-3 cells transduced with the R%SGGEAKNI TCR, while irrelevant peptides did not (Fig. 5, B and C). Coincidentally, a representative TCR from RKQG%E responded to “LDEISTNIRQAGVQY,” and the related peptide “EISTNIRQAGVQYSRADEEQ” from subpool 13, CFP10-derived peptides distinct from the R%SGGEAKNI epitope (Fig. 5 C). Interestingly, the shorter “ISTNIRQAGVQYSRADEE” peptide was recently described to be presented by Mtb-infected MDCs, suggesting CFP10-specific clonotypes were generated in response to infection *in vivo* (Leddy et al., 2024, *Preprint*).

Next, we determined the antigen specificities of representative TCRs containing the S%SGTKYNE, SSPGQGG%NYG, and %MPE motifs. For the S%SGTKYNE TCR, individual peptides from subpool 3 revealed CD69 expression exclusively by TCRβ^+^ cells in response to the “MSFVTTQPEALAAA” peptide common to PE12 (Rv1172c), PE18 (Rv1788), and PE19 (Rv1791), secreted substrates of the Esx5 T7SS (Sayes et al., 2016) (Fig. 5, D and E). The SSPGQGG%NYG TCR responded to “VDLAKSLRIAAKIYS,” a peptide from EspC (Rv3615c), a substrate of the Esx1 T7SS, which is cosecreted with EspA and which is also essential for the secretion of CFP10 and ESAT6 (Millington et al., 2011; Garces et al., 2010) (Fig. 5 F). The %MPE TCR responded to “AAFQGAHARFVAAAA” and “AAFQAAHARFVAAAA,” peptides from EsxS and EsxG, respectively (Fig. 5 G). The latter, an EsxG peptide, was also found to be presented by Mtb-infected DCs (Leddy et al., 2024, *Preprint*). GLIPH2 groups containing TCRs linked to recognition of infected macrophages, and confirmed to be specific for Mtb antigens, expressed CDR3α and CDR3β chain homology (Fig. 5 I). However, TCRs from RKQG%E and SLRT%ET, which were linked to MTB300 or lysate responses only (Fig. 4 C), showed reduced or absent CDR3α consensus sequences (Fig. 5 I). These results reveal the Mtb antigens targeted by TCRs from the GLIPH2 group linked to recognition of infected macrophages or Mtb lysate.

**Figure 6.**
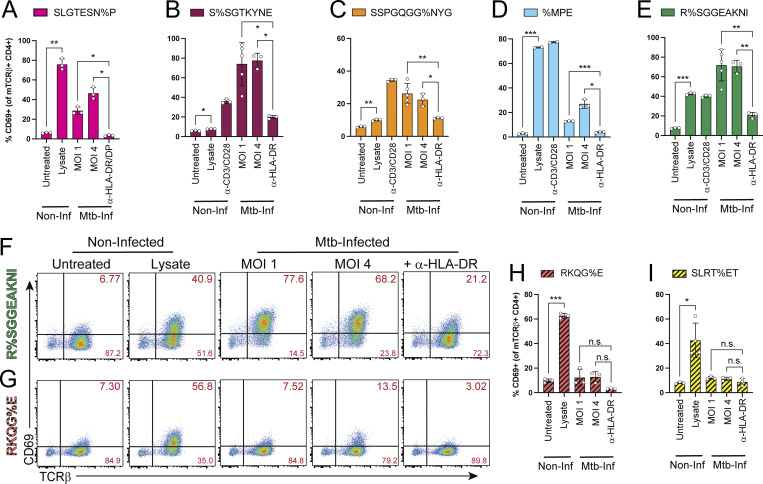
**TCRs from Mtb-specific GLIPH2 groups vary in their capacity to recognize infected macrophages. (A–E)** Bar graphs of mean (±SD) %CD69^+^ cells by flow cytometry (gated on CD4^+^ TCRβ^+^ Live-Dead^Lo^) for SKW-3 cells transduced with representative TCRs from each of the indicated GLIPH2 groups 18 h after coculture with macrophages infected at MOI 1 or 4 (± α-HLA-DR/DP/DQ blockade), or left uninfected ± treatment with lysate or anti-CD3/CD28 bead stimulation. **(F and G)** Representative flow cytometry plots of CD69 and TCRβ expression of CD4^+^ SKW-3 cells transduced with the (F) R%SGGEAKNI or (G) RKQG%E TCRs in response to Mtb-infected or lysate-treated macrophages in separate experiments. **(H and I)** Bar graphs of mean (±SD) CD69 expression (of CD4^+^ TCRβ^+^) for the (H) RKQG%E and (I) SLRT%ET TCRs in response to infected macrophages. Data are representative of two to three independent experiments containing two to five replicates per condition. Statistical significance was determined using a Welch one-way ANOVA and Dunnett’s T3 post hoc test corrected for multiple comparisons; *P < 0.05, **P < 0.01, ***P < 0.001, n.s., not significant.

Finally, SKW-3 cells expressing each TCR were tested for their capacity to recognize Mtb-infected macrophages or lysate. 1 day after infection of MDMs at MOI 1 or 4, or treatment with lysate, TCR-transduced SKW-3 cells were added for 16–18 h and CD69 expression was assessed by flow cytometry. TCRs cloned from R%SGGEAKNI (specific for CFP10), S%SGTKYNE (PE12/PE18/PE19), SSPGQGG%NYG (EspC), %MPE (EsxG/EsxS), and SLGTESN%P (EspA) all responded to infected macrophages though with varying magnitudes of activation (Fig. 6, A–F). The %MPE TCR-transduced cells (Fig. 6 D) displayed the lowest proportion activated, while S%SGTKYNE (Fig. 6 B) and R%SGGEAKNI (Fig. 6, E and F) had the highest proportions activated in response to Mtb-infected macrophages, compared with lysate controls. Activation was significantly reduced with MHC-II blockade, indicating it was mediated by TCR- pMHC engagement. As expected from the memory CD4^+^ T cell data, TCRs from RKQG%E (CFP10-specific) and SLRT%ET (yet-to-be defined peptide in Mtb lysate) showed little to no activation in response to infected macrophages despite responding to lysate (Fig. 6, G–I). While the efficiency of activation differed between clonotypes, these data validate the recognition of infected macrophages, or lack thereof, observed for primary memory CD4^+^ T cells with TCRs from multiple GLIPH2 groups.

### Single-cell transcriptomics reveals distinct phenotypes of memory CD4^+^ T cell responses to infected macrophages

**Figure S3.**
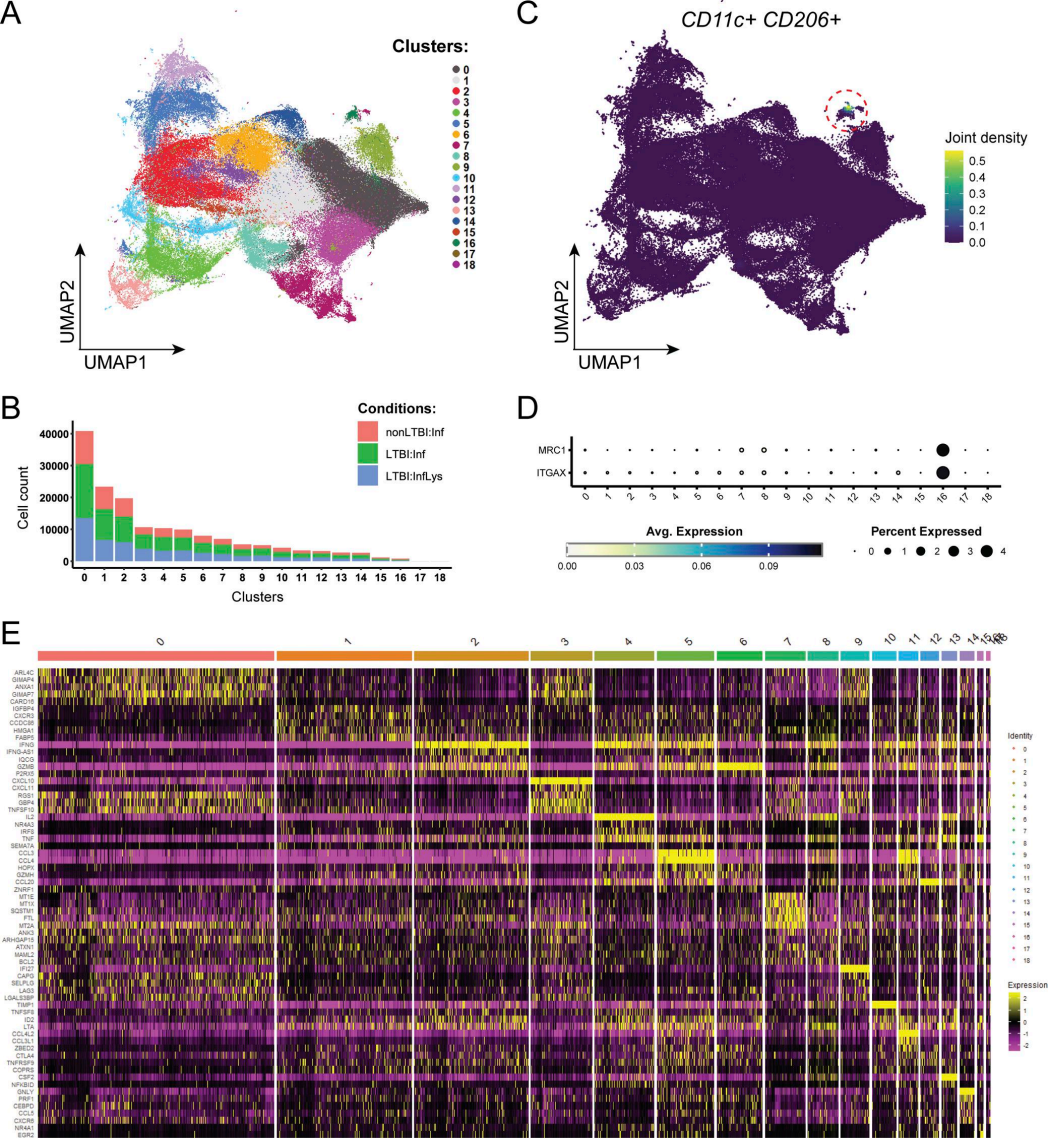
**Cellularity and monocytic contamination as quality control metrics after integration.** Related to Fig. 7. **(A)** UMAP plot showing unbiased clustering of the CD4^+^ populations of 6 non-LTBI (6 donors) and 12 LTBI samples (7 donors) after quality control and integration, and prior to monocytic contamination removal. **(B)** Bar plot depicting cell numbers in each cluster per experimental condition. **(C)** UMAP plot showing joint density estimation for plot for CD11c and CD206 transcripts in the integrated dataset. **(D)** Dot plot showing the average expression and percentage of cells expressing CD11c and CD206 transcripts in each cluster. **(E)** Heatmap showing top five DEGs in the integrated dataset for each cluster prior to removal of low-quality clusters, e.g., clusters 16, 17, and 18.

**Figure 7.**
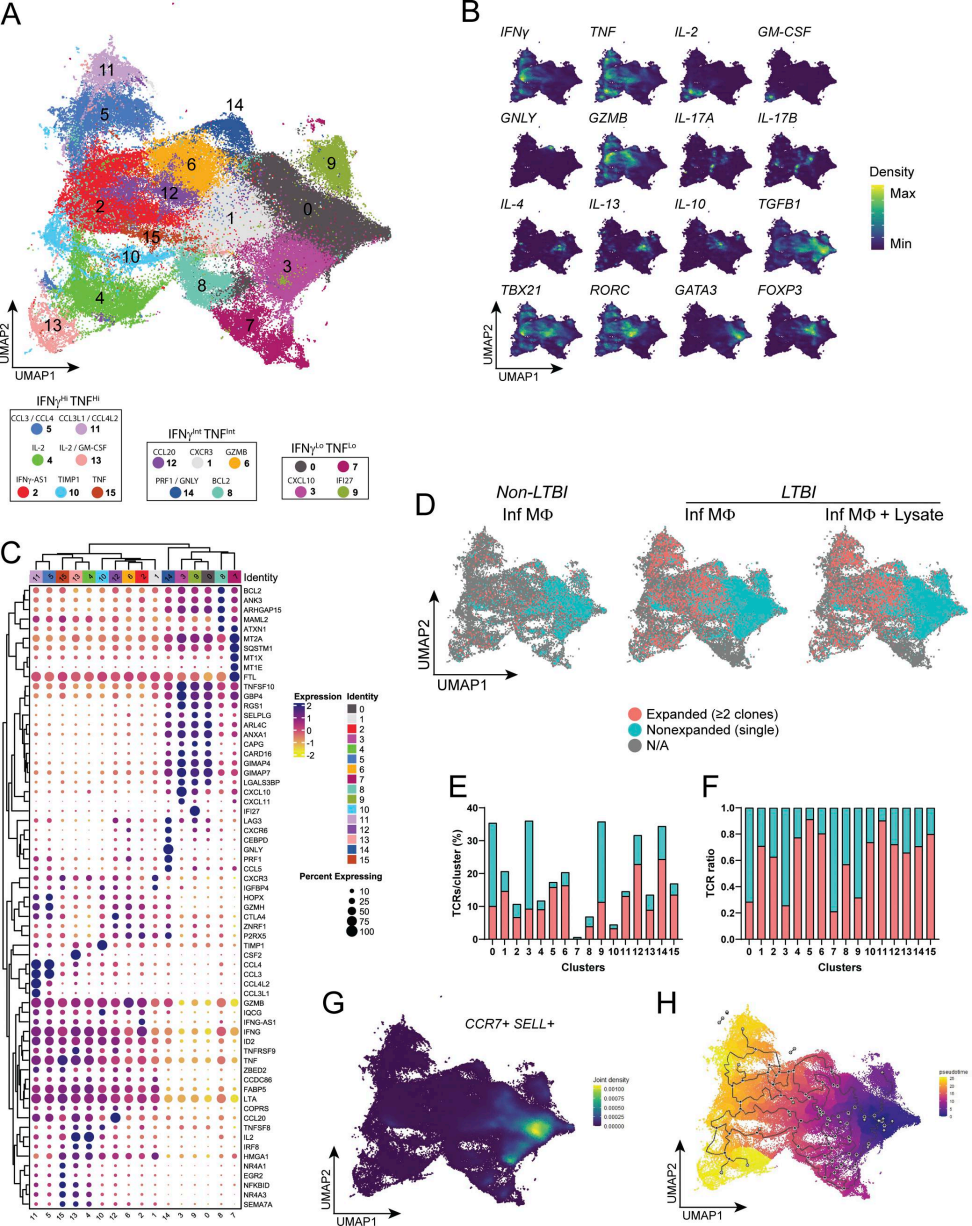
**Single-cell transcriptomics reveals distinct phenotypes of memory CD4** ^**+**^ **T cell responses to infected macrophages. (A)** UMAP visualization plot including the Louvain clustering of 157,462 cells after integration and quality control from 7 LTBI participants (12 samples, AIM^+^ memory CD4^+^ T cells in response to infected macrophages ± lysate) and 6 non-LTBI participants (6 samples, AIM^+^ memory CD4^+^ T cells in response to infected macrophages). Top DEGs for each cluster are listed below plot, grouped by IFNγ and TNF expression. **(B)** Kernel density estimation of selected T helper subset genes projected onto the UMAP plot. Density values were reduced to max/min scale. **(C)** Heatmap with hierarchical clustering (left) showing top five DEGs for each cluster, conserved across treatment groups. **(D)** Split UMAP plots for experimental groups showing mapping of all TCRs. Expanded (≥2 copies) and nonexpanded (single) TCR clonotypes are shown in red and blue, respectively. **(E and F)** Representative stacked bar plots showing (E) percent clonally expanded versus nonexpanded TCRs of total TCRs sequenced from LTBI participant samples, and (F) ratio of expanded and nonexpanded TCRs normalized to each cluster’s total cell number. **(G and H)** Joint density estimation plot for (G) *CCR7* and *SELL* transcripts and (H) pseudotime trajectory (rooted in cells with the highest expression of *CCR7* and *SELL*), projected onto the UMAP plot. Cell fates (gray circles), transition states (black circles), and proximity to (purple) and remoteness from (yellow) the root are indicated.

To explore the effector responses of memory CD4^+^ T cells, we performed scRNAseq 16–18 h after *ex vivo* coculture with infected macrophages (± treatment with lysate) in parallel with scTCRseq (Fig. 1 A). An integrated dataset was generated from 157,462 high-quality CD4^+^ T cells using Seurat v5 (Satija et al., 2015). 19 unsupervised cell clusters were generated and visualized using Uniform Manifold Approximation and Projection (UMAP) (Fig. S3 A). The three smallest clusters were removed for quality control due to the simultaneous expression of macrophage markers *ITGAX* (CD11c) and MRC-1 and the lack of TCRs, or low cell numbers (see Materials and methods) (Fig. S3, B–D). The 16 remaining clusters were then analyzed (Fig. 7 A).

**Figure S4.**
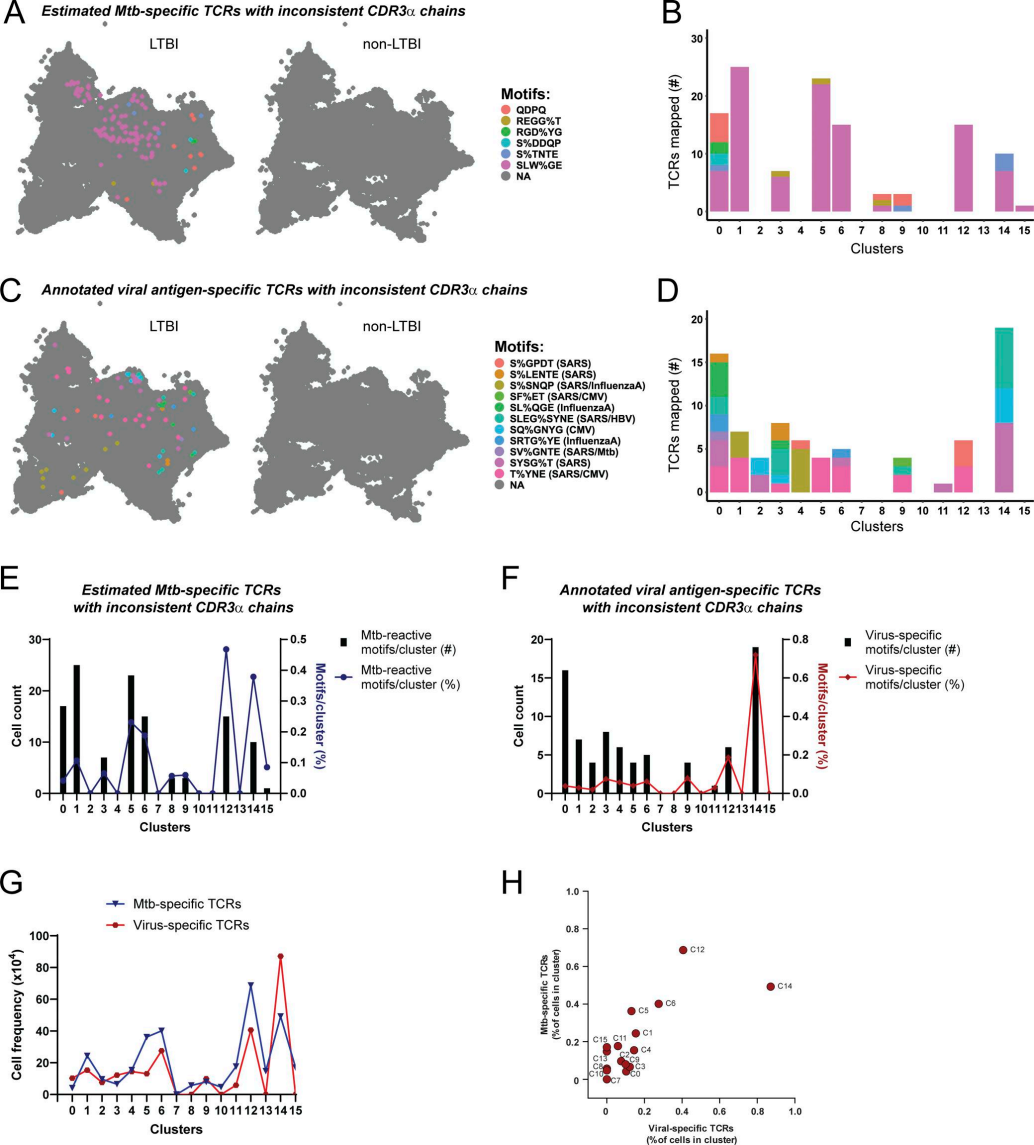
**TCRs with inconsistent CDR3α chains map to clusters with nonspecific CD4** ^**+**^ **T cell responses.** Related to Fig. 8. **(A and B)** (A) UMAP plot with split view (based on the participant’s LTBI status) with mapping of estimated Mtb-specific TCRs from listed GLIPH2 groups containing inconsistent CDR3α homology in response to Mtb-infected macrophages, and (B) stacked bar plots showing their numbers per cluster. **(C and D)** (C) UMAP plots with mapping of TCRs annotated as viral antigen–specific but with inconsistent CDR3α homology from listed GLIPH2 groups and (D) stacked bar plots showing their numbers per cluster. **(E and F)** (E) Total copy numbers (left axis) and percentage (right axis) ([clone count/total cells per cluster] × 100) of cells per cluster mapping TCRs linked to a response to infected macrophages from listed GLIPH2 groups containing inconsistent CDR3α homology from known or estimated Mtb-specific GLIPH2 groups, and (F) for annotated viral antigen–specific TCRs. **(G and H)** (G) Overlaid line graph and (H) correlation plot of percent T cells per cluster that mapped TCRs from all 37 GLIPH2 groups estimated to be Mtb-specific (blue) and viral antigen–specific (red).

To define the phenotypic composition of each cluster, we used the combination of gene density visualization for CD4^+^ T cell effector genes and evaluation of the top five conserved differentially expressed genes (DEGs) for each cluster (Fig. 7, B and C; and Fig. S4 E). The expression of *IFNG* and *TNF*, canonical Th1 cytokines, was observed in 12 of 16 clusters, along with the high expression of *CXCR3*, together with *LTA* (lymphotoxin-α), *GZMB* (granzyme B), *ID2*, and *FABP5* (Fig. 7 C and Fig. S3 E). *IL-2* expression was prominent in clusters 4 and 13, with cluster 13 also expressing *CSF2* (GM-CSF). *CCL3* and *CCL4*, chemokines associated with immune cell recruitment (Castellino et al., 2006), were preferentially expressed in clusters 5 and 11, and *CCL3L1* and *CCL4L2* were expressed in cluster 11. Cluster 6 contained the highest *GZMB* expression, cluster 12 was enriched for *CCL20*, and the top DEGs for cluster 14 were the *PRF1* (perforin) and *GNLY* (granulysin), indicating cytotoxic CD4^+^ T cell responses (Fig. 7, A and C). Cluster 1 was enriched for CXCR3 expression, but also contained the expression of several other cytokines, including *IL10*, *IL17A*, and *IL17F* (Fig. 7, A–C). While clusters 0, 3, 7, and 9 expressed little to no *IFNG* or *TNF*, they instead expressed the type I and II IFN response genes *IFI27* and *CXCL10*, respectively, along with *TGFB1* and *IL10* (Fig. 7 B). Together, these data indicate heterogeneous CD4^+^ T cell effector responses to Mtb-infected macrophages.

### CDR3 mapping distinguishes the effector functions of Mtb-specific TCR clonotypes

To examine the functions of clonally expanded CD4^+^ T cells in response to infected macrophages (± treatment with lysate), we first visualized the mapping of expanded TCRs (≥2 copies) compared with nonexpanded TCRs (1 copy) from LTBI and non-LTBI participants (Fig. 7 D). Among all groups, single TCR clonotypes were localized within clusters 0, 3, 7, and 9 (Fig. 7, D–F), which lacked the expression of *IFNG*, *TNF*, and *LTA* (Fig. 7, A–C). The transcriptional profiles in clusters 0 and 3, including the co-expression of *CCR7* and *SELL* (CD62L), suggested minimal differentiation and a central memory-like T cell phenotype (Fig. 7 G). Pseudotime projection rooted within clusters 0 and 3 indicates the most differentiated T cells to reside within clusters 4 and 13 followed by clusters 11 and 5 (Fig. 7, G and H). These high-differentiated CD4^+^ T cell clusters mapped the greatest proportions of clonally expanded TCRs (Fig. 7, D–F).

**Figure 8.**
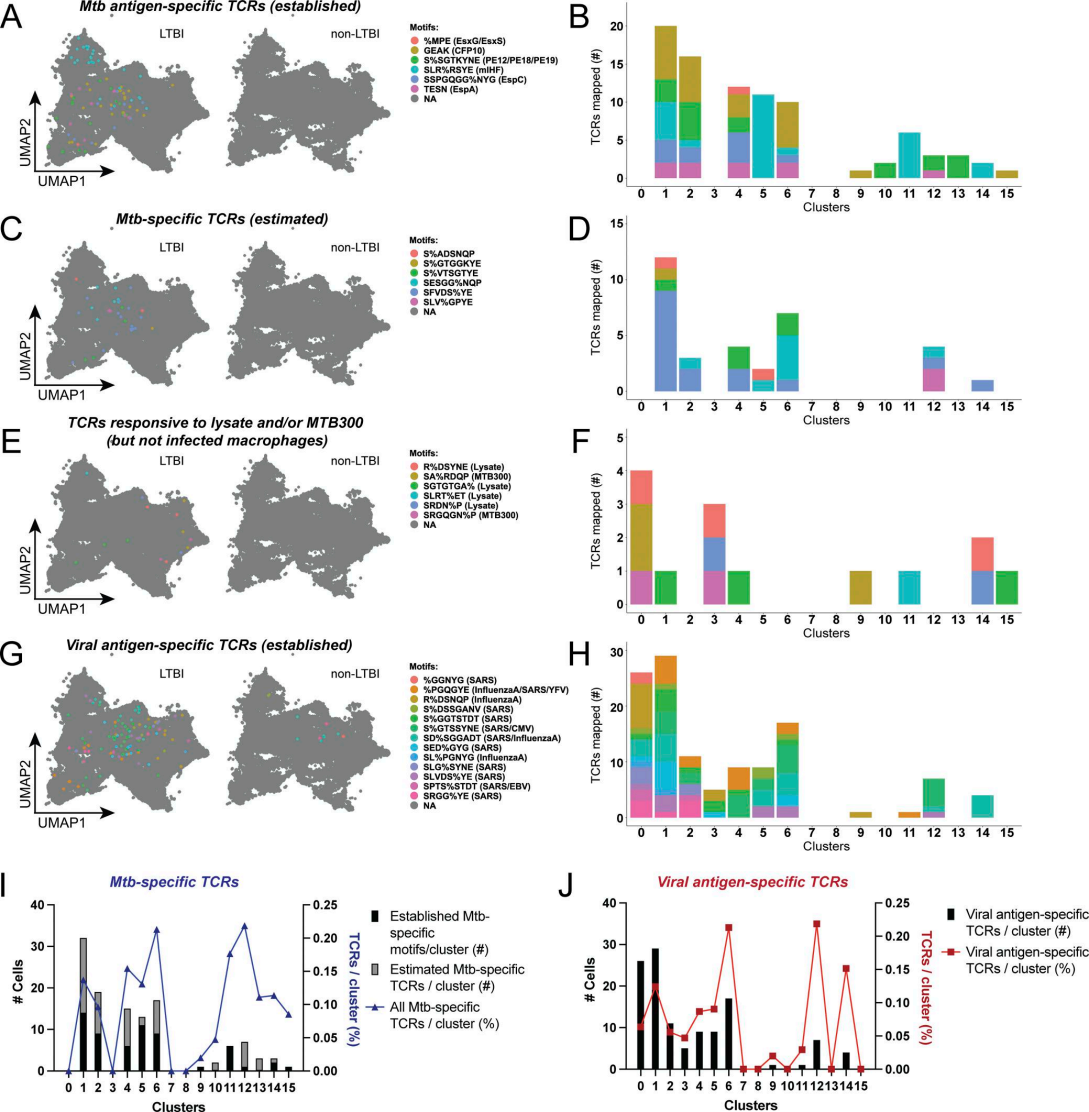
**CDR3 mapping distinguishes the effector functions of Mtb-specific TCR clonotypes. (A and B)** (A) UMAP plot with split view (based on cells from LTBI vs. non-LTBI participants) with mapping of TCRs from listed GLIPH2 groups (motifs) established as Mtb antigen–specific, and (B) stacked bar plot showing numbers of αβTCRs from each GLIPH2 group (color) per cluster. **(C and D)** (C) UMAP plots mapping αβTCRs from GLIPH2 groups estimated to be Mtb-specific, and (D) stacked bar plots showing numbers of TCRs per cluster. **(E and F)** (E) UMAP plots mapping TCRs from listed GLIPH2 groups responsive to MTB300 and/or lysate stimulation, but not infected macrophages, and (F) stacked bar plots showing numbers of TCRs per cluster. **(G and H)** (G) UMAP plots mapping annotated viral antigen–specific TCRs (in IEDB) from listed GLIPH2 groups, and (H) stacked bar plots showing numbers of TCRs per cluster. **(I and J)** (I) Total copy numbers (left axis) and percentage (right axis) of cells per cluster mapping TCRs ([clone count/total cells per cluster] × 100) from established or estimated Mtb-specific GLIPH2 groups that recognized infected macrophages and (J) for annotated viral antigen–specific TCRs.

To explore the relationship between CD4^+^ T cell phenotypes and their capacity to recognize infected macrophages, we mapped TCRs from relevant GLIPH2 groups onto single-cell transcriptomics data. In addition to homologous CDR3β chains, we categorized GLIPH2 groups as “consistent,” if displaying high CDR3α homology (differing by ≤2 amino acids), and “inconsistent” when displaying greater variation (Fig. S4 A). Focusing on TCRs from consistent GLIPH2 groups (Fig. 4 H), those previously established as Mtb-specific (Fig. 8, A and B), and those likely to be Mtb-specific (Fig. 8, C and D) mapped to clusters 1, 2, 4, 5, 6, and 10–15. Although fewer clonotypes responded only to MTB300 and/or lysate, they distinctly mapped to clusters 0, 3, and 14 (Fig. 8, E and F). We also evaluated GLIPH2 groups containing at least one TCR with 100% CDR3β homology to viral antigen–specific TCRs annotated in the IEDB. Similar to GLIPH2 groups with inconsistent CDR3α homology (Fig. S4, A–D), clusters 0, 1, 3, 6, 12, and 14 were enriched for viral antigen–specific TCRs (Fig. 8, G and H). TCR mapping was also represented as the percentage of total cells per cluster to reduce TCR assignment bias due to differences in cell numbers per cluster (Fig. 8, I and J; and Fig. S4, E and F). These data highlight that in response to infected macrophages, Mtb-specific CD4^+^ T cells express distinct effector functions when compared to viral antigen–specific TCR clonotypes.

### Mtb-specific CD4^+^ T cells express signature effector genes in response to infected macrophages

**Figure 9.**
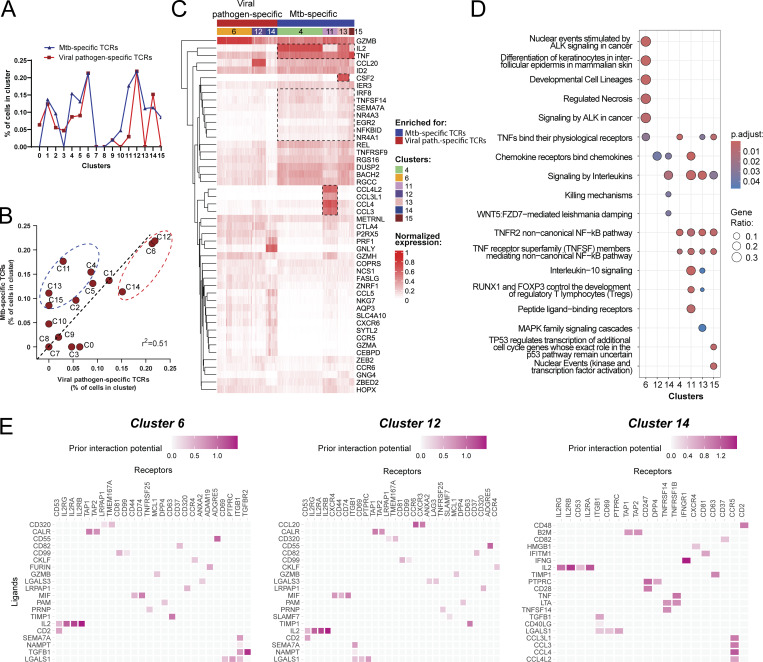
**Mtb-specific CD4** ^**+**^ **T cells express signature effector genes in response to infected macrophages. (A and B)** (A) Overlaid line graph and (B) correlation plot of percent T cells per cluster that mapped Mtb-specific (blue) and viral pathogen-specific (red) TCRs. Linear regression (black dashed line) and Pearson’s correlation coefficient squared (r^2^) are shown. Red and blue dashed circles identify UMAP clusters with highest enrichment of Mtb-specific and viral pathogen-specific TCRs, respectively. **(C)** Heatmap showing top 10 DEGs normalized to the individual maximum expression for listed clusters. Gene expression patterns common to cluster enriched for Mtb-specific TCRs are outlined. **(D)** Dot plot showing Reactome pathway overrepresentation analysis using the lists of genes with Log_2_FC > 1 for UMAP clusters 4, 6, and 11–15. The list of top seven most significant pathways is arranged based on the GeneRatio (number of input genes associated with a Reactome term/total number of input genes). **(E)** Summary of receptor–ligand pairs identified by NicheNet analysis, estimating the cell–cell communication (prior interaction potential) between “sender” clusters 4, 11, 13, or 15 (combined) and “receiver” cluster 6 (left), cluster 12 (middle), and cluster 14 (right).

**Figure S5.**
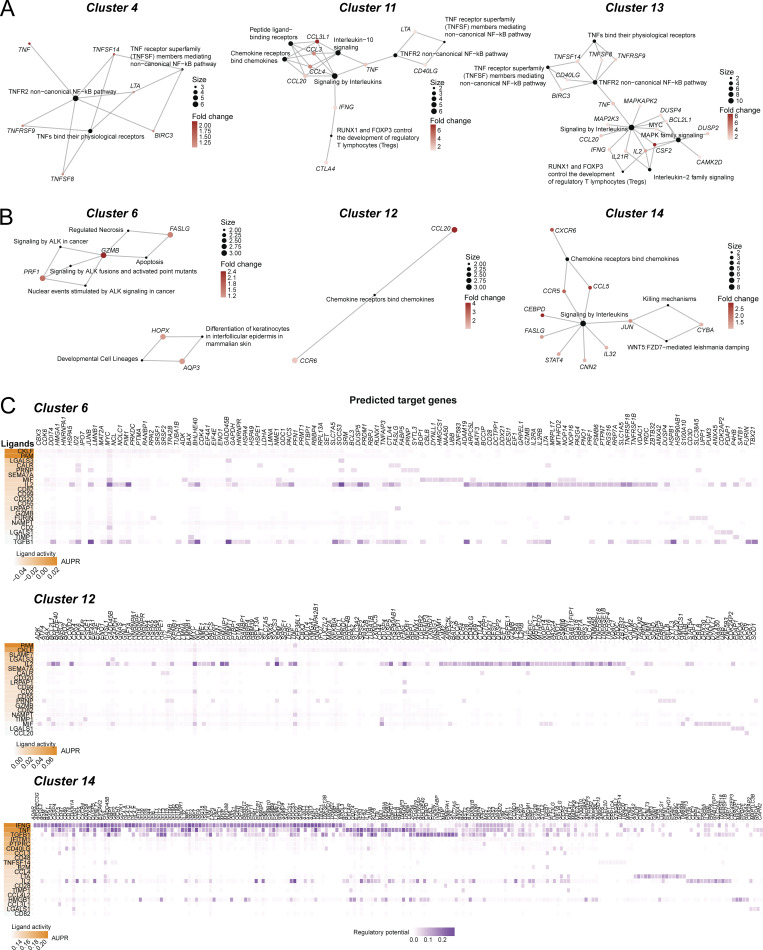
**Mtb-specific TCR clonotypes communicate with other memory T cells to control infection.** Related to Fig. 9. **(A and B)** Network plots illustrating Reactome pathways with top DEGs labeled (A) for clusters 4, 11, 13, and 15, and (B) for clusters 6, 12, 14. **(C)** Heatmaps showing downstream signaling genes estimated to be linked to cell–cell communication between ligands expressed by clusters 4, 11, 13, or 15 (combined) and T cells in clusters 6 (top), 12 (middle), and 14 (bottom). The AUPRC was used to rank the ligand activity of senders on responders (left). AUPRC, area under the precision–recall curve.

To determine the profiles of Mtb-specific recognition of infected macrophages, we compared the frequencies of Mtb and viral antigen–specific TCRs from corresponding GLIPH2 groups that mapped to each cluster. Clusters 4, 11, 13, and 15 were proportionally enriched with Mtb-specific TCRs (Fig. 9, A and B). Interestingly, these same CD4^+^ T cell clusters were also found to express the most differentiated phenotype (Fig. 7 H). Clusters 6 and 12 mapped the greatest frequencies of both Mtb and viral antigen–specific TCRs as a proportion of total cells, while cluster 14 contained the highest proportion of viral antigen–specific TCRs (Fig. 9 B). Although our analysis focused on consistent GLIPH2 groups, a similar distribution was observed if TCRs from all 29 GLIPH2 groups were included (Fig. S4, G and H). Clusters enriched with Mtb-specific TCRs included the preferential expression of *CCL3*, *CCL4*, *CCL3L1*, and *CCL4L2* (cluster 11), *IL-2* (clusters 4 and 13), *GM-CSF* (cluster 13), and *IRF8*, *TNFSF14*, *SEMA7A*, *NR4A3*, *NR4A1*, and *NFKBID* (clusters 4, 13, and 15) (Fig. 9 C). To examine the pathways involved, we performed Reactome pathway overrepresentation analysis using the top DEGs (Log_2_FC > 1) for clusters 4, 6, and 11–15. Despite containing distinct gene expression signatures, all clusters enriched for Mtb-specific TCRs shared a biological theme centered around noncanonical NFκB pathway signaling mediated by TNF superfamily members or TNFR2 signaling (Fig. 9 D and Fig. S5 A). In contrast, pathways for clusters 6 and 14 were dominated by cytotoxic responses (Fig. 9 D and Fig. S5 B). Together, these data identify pathways common and unique to clusters enriched for Mtb-specific TCR clonotypes.

Finally, to test the hypothesis that Mtb-specific clonotypes express factors that contribute to the recruitment and activation of other memory T cells to control infection, receptor–ligand pairs were evaluated by analysis with NicheNet (Browaeys et al., 2020). Using the DEGs from T cells enriched with Mtb-specific TCRs (clusters 4, 11, 13, and 15), as potential “sender” ligands, we identified IL-2 signaling through CD53, IL-2Rα, IL-2Rβ, or IL-2Rγ, as the ligand–receptor pair common to clusters 6, 12, and 14 with dominant downstream signaling patterns (Fig. 9 E and Fig. S5 C). CCL3, CCL4, CCL3L1, and CCL4L2 signaling through CCR5, IFNγ signaling through IFNγR1, TGFβ signaling through ITGB1, and TNF and LTα signaling through TNFR2 also represented candidate ligand–receptor interactions with cluster 14 (Fig. 9 E and Fig. S5 C). Each of these cytokine and chemokine ligands represented top DEGs preferentially expressed by the clusters enriched with Mtb-specific TCRs, except for TGFβ, which is expressed by T cells in clusters 0 and 3 (Figs. 7, A–C; and Fig. 9 C) and infected MDMs (Gail et al., 2023). These data indicate that top cytokines and chemokines expressed by Mtb-specific CD4^+^ T cells have the potential to provide recruitment and activation signals to other memory T cells in response to Mtb infection.

## Discussion

In this study, we describe the first analysis of human memory CD4^+^ T cell responses to autologous macrophages infected with virulent Mtb using TCR sequencing and transcriptomics at a single-cell level. The majority (73–90%) of memory CD4^+^ T cells from 10 individuals with LTBI were found to recognize infected macrophages, but a subset responded only when exogenous antigens were added. We focused on expanded TCRs (≥2 copies) containing CDR3 motifs overrepresented among multiple individuals. We used GLIPH2 to segregate TCRs specific for other pathogens via the use of control peptide stimulations or those annotated as specific for viral antigens in the IEDB. This workflow resulted in 29 GLIPH2 groups containing TCRs with shared CDR3β motifs predicted to be Mtb-specific. TCRdist3 clustered most of the same TCRs into narrow meta-clonotypes, each containing fewer TCRs with representation from fewer individuals. Since GLIPH2 allows TCRs to participate in more than one group (Huang et al., 2020), relevant TCRs are not necessarily eliminated when less statistically robust groups are removed resulting in broader motifs, each containing more TCRs and individuals represented, but also risks grouping unrelated TCRs. The use of infected macrophage and peptide stimulations, together with TCR clustering, served to identify Mtb-specific clonotypes from individuals with stable LTBI.

Of the TCRs cloned and screened for cognate peptides, all were found to be specific for secreted Mtb antigens, which are T7SS substrates of the Esx1 (EspA, EspC, and CFP10), Esx3 (EsxG, EsxS), and Esx5 (PE12, PE18, PE19) loci. Most are known immunodominant antigens shown to be essential for virulence *in vivo* (Gröschel et al., 2016). Interestingly, Mtb strains containing deletions in secreted Esx1, Esx3, and Esx5 genes are attenuated for growth *in vivo* (Sayes et al., 2016; Wang et al., 2022; Guinn et al., 2004; Garces et al., 2010). In fact, persistent activation of CD4^+^ T cells specific for ESAT6, a virulence factor cosecreted with and codependent on CFP10, EspA, and EspC, was observed in the mouse model of TB (Moguche et al., 2017; Garces et al., 2010). The enrichment of secreted T7SS substrates is remarkable considering representation of >90 diverse antigens in the MTB300 peptide megapool (Lindestam Arlehamn et al., 2016). We posit that serving an essential pathogenesis role makes these Mtb antigens effective targets of protective memory CD4^+^ T cells.

Although a majority of Mtb-specific TCRs were linked to recognition of infected macrophages, at least 10% responded only to MTB300 peptides or lysate. Recent data from the mouse model of TB also found that not all Mtb-specific T cells generated during TB are able to recognize infected macrophages (Yang et al., 2018; Patankar et al., 2020). Our results show that TCRs containing the R%SGGEAKNI motif, which efficiently recognized infected macrophages, and the RKQG%E motif, which did not, both targeted the CFP10 antigen but were specific for distinct peptides. Since cognate peptides for both TCRs were shown by immunopeptidomics to be presented by Mtb-infected cells (Leddy et al., 2024, *Preprint*), it was surprising the RKQG%E TCR did not efficiently recognize infected macrophages. One explanation is a difference between the processing and presentation of peptides by macrophages vs. DCs upon infection with Mtb. Interestingly, the more efficient cognate peptide for the RKQG%E TCR, LDEISTNIRQAGVQY, was not the one found to be presented by Mtb-infected DCs. Differences in peptide abundance, processing within distinct APC subsets, and TCR-pMHC binding interactions (e.g., functional avidity, peak bond lifetime) could each contribute recognition efficiency (Faust et al., 2023; Ewanchuk and Yates, 2018; Carpenter et al., 2016). Interestingly, the TCRs that did not efficiently recognize infected macrophages were from GLIPH2 groups containing little or no CDR3α homology, suggesting reliance on the CDR3β chain for recognition. Such chain centricity has been linked to cross-reactive and autoreactive TCRs where crystallography showed tilted TCR-pMHC binding topology (Sethi et al., 2013). The triggering of such TCRs may require greater concentrations of Mtb peptides than are presented by infected macrophages. Virulence factors, such as EsxH, could play a role by limiting antigen presentation in infected but not noninfected cells (Portal-Celhay et al., 2016). Finally, certain antigens could elicit expansion of clonotypes that do not recognize infected macrophages later during *in vivo* infection due to bacterial regulation of antigen expression *in vivo* (Bold et al., 2011; Moguche et al., 2017). Therefore, distinct TCRs targeting peptides processed and presented by infected macrophages, but differ in binding strength and kinetics, could account for a subset of the repertoire that lacks recognition of infected macrophages, either in addition to or instead of “hidden” antigens.

Based on recently published work, differences in antigen processing and presentation between DCs in MLNs and macrophages in the lung could account for the priming of T cells that do not recognize infected macrophages (Ewanchuk and Yates, 2018). Upon infection, DCs in MLNs likely present more Mtb peptides than infected macrophages and therefore prime a broad repertoire of antigen-specific T cells. Supporting this hypothesis, differences in phagocyte NADPH oxidase (NOX2) activity were shown to directly affect thiol transferase and cysteine cathepsin activity, and therefore the repertoire of peptides presented in the context of MHC-II (Allan et al., 2014; Rybicka et al., 2010). The regulation of these cathepsins by NOX2 is likely more important to epitope preservation in lung macrophages where the consequences of activating T cells and promoting inflammation that affect gas exchange can be severe. It is also logical that to fight infection, the immune system would evolve ways to recruit and activate other memory T cells that depend on antigen-specific responses, while preventing the activation of other T cells in the surrounding tissue. Individuals with recent Mtb infection and IGRA conversion represent a state of higher risk for active TB, while exposed individuals who remain IGRA-negative or have long-standing stable IGRA-positivity are associated with greater protection from active TB (Andrews et al., 2012; Kim et al., 2024). We posit that individuals who possess a greater risk of active TB have a higher proportion of memory CD4^+^ T cells that do not efficiently recognize infected macrophages.

T cells sharing similar CDR3 motifs often exhibit overlapping antigen specificity, transcriptional profiles, and cell fate, particularly in the context of infectious diseases (Chen et al., 2023; Wang and Ji, 2023, *Preprint*). By mapping TCRs to their gene expression profiles, we discovered Mtb-specific responses linked to recognition of infected macrophages preferentially co-express *IFNG*, *TNF*, *IRF8*, *TNFSF14*, *SEMA7A*, *NR4A3*, *NR4A1*, *NFKBID*, and *CCL3*, *CCL4*, *CCL3L1*, and *CCL4L2* (Fig. 8 C, cluster 11), or *IL2* and *CSF2* (GM-CSF) (Fig. 8 C, cluster 13). Cluster 15 (highest *TNF* expression), cluster 5 (expressing *CCL3* and *CCL4*), and cluster 4 (expressing *IL2*) were also enriched for Mtb-specific TCRs. Of these, clusters 11 and 13 contained the highest enrichment for Mtb-specific TCRs and were the most differentiated T cell clusters based on trajectory analysis. The highest frequencies of all clonally expanded TCRs, and those annotated as viral antigen–specific, mapped to clusters 6, 12, and 14, suggesting nonspecific bystander T cell activation. These clusters were enriched for cytotoxic gene expression, including *GZMB*, *PRF1*, *GNLY*, and *CCL20*. The nonexpanded TCRs were enriched among clusters 0, 3, 7, and 9, which expressed central memory-like phenotype, regulatory functions, and IFN response genes, indicating a second program of bystander T cell activation. Given their enrichment with Mtb-specific TCRs, and their distinct and differentiated transcriptomics profiles, we conclude the effector phenotypes of clusters 4, 11, 13, and 15 represent Mtb-specific memory CD4^+^ T cell recognition of infected macrophages. Validation that these effector programs are linked to restriction of bacterial growth will be needed to confirm their relevance to protective CD4^+^ T cell responses.

We initially sought to compare Mtb-specific CD4^+^ T cell responses that did or did not recognize infected macrophages. However, we were struck by the large numbers of viral antigen–specific TCR clonotypes activated in response to infection. We considered both cross-reactivity due to molecular mimicry and nonspecific bystander activation of memory T cells. Since humans contain only ∼10^12^ total and ∼10^8^ unique TCR clonotypes available to recognize the >10^15^ foreign peptides (Arstila et al., 1999; Mason, 1998; Sewell, 2012), bystander activation and cross-reactivity of immunological memory extend protection of antigen-specific T cells across multiple infectious diseases (Su et al., 2013; Kundu et al., 2022; Bartolo et al., 2022). Shared epitopes between viral pathogens and Mtb have not been identified. Exposure to environmental nontuberculous mycobacteria (NTM) by study participants would likely generate T cells cross-reactive to homologous NTM proteins (Prasad et al., 2013; Shah et al., 2019). Yet, we observed highly expanded clonotypes in many different participants’ samples that either responded to control peptide stimulation or were already annotated in the IEDB as specific for SARS-CoV-2, influenza A, CMV, or EBV antigens. These features suggest bystander activation rather than cross-reactivity.

Bystander activation of CD8^+^ and CD4^+^ T cells during acute viral infection, or after vaccination, respectively, is well described (Ehl et al., 1997; Tough et al., 1996; Doisne et al., 2004; van Aalst et al., 2017; Di Genova et al., 2006). T cell activation that does not involve TCR-pMHC interactions can occur via cytokines, including IL-2 and IL-15 signaling (Bangs et al., 2006), IL-12 and IL-18 signaling (Srinivasan et al., 2007), or via costimulatory receptors such as 4-1BB (Reithofer et al., 2021) and NKG2D ligation (Balint et al., 2024), or combinations thereof (van Aalst et al., 2017). Our results extend earlier observations to TB, where we find antimicrobial functions among clonally expanded bystander memory CD4^+^ T cells but regulatory functions (e.g., IL-10, TGFβ production) among nonexpanded bystander TCR clonotypes. In addition to their antimicrobial functions, our data suggest Mtb-specific CD4^+^ T cells participate in the recruitment and activation of other memory T cells, which may underpin the observed expression of multiple effector T cell programs. The cell–cell interaction analysis suggests antigen-specific CD4^+^ T cells interact with bystander-activated CD4^+^ T cells through IL-2, TNF, IFNγ, and chemokines (CCL3, CCL4, CCL3L1, CCL4L2). Therefore, we infer that bystander T cell activation requires signals from antigen-specific CD4^+^ T cells, as well as infected macrophages, and that antimicrobial immune responses could be amplified from small numbers of Mtb-specific T cells.

The enormous diversity of HLA alleles and TCRs in humans is a major barrier to identifying pathogen-specific T cell responses. Diversity in the TCR repertoire is driven by variation in HLA alleles between and within individuals, and by differences in the repertoires of Mtb peptides presented to T cells upon infection. TCR grouping algorithms like GLIPH2 identify clonotypes that contain homologous CDR3β motifs present in samples from multiple individuals who share a common HLA allele and were shown to have specificity for the same peptide epitope. Using lists of Mtb-reactive TCRs from both Cleveland and South African participants, four GLIPH2 groups (SLR%RSYE, SPGTESN%P, RA%GGEAKNI, and SLGTESN%P) were identical or homologous to those found in two published LTBI studies (Glanville et al., 2017; Huang et al., 2020). We also identified GLIPH2 groups (R%SSGEAKNI, ALFG, and SRDN%P) identical to those from “nonprogressors” in the Adolescent Cohort Study (Musvosvi et al., 2023), suggesting our local study participants with stable LTBI are enriched with protective TCRs. Preferential activation and expansion of memory CD4^+^ T cells that recognize infected macrophages may explain protective T cell responses in stable LTBI.

### Limitations of the study

Our study is the first to examine primary human memory CD4^+^ T cell recognition of Mtb-infected macrophages using single-cell transcriptomics, yet it has limitations. An important limitation is the number of participants with LTBI. Although our objective was to conduct a proof-of-concept study of the capacity of human memory CD4^+^ T cells to recognize Mtb-infected macrophages, the small sample size limits the generalizability of our findings across diverse populations, as well as the ability to perform HLA association analyses. However, the trade-off was the ability to assess multiple conditions using high-throughput scRNAseq, which, although costly, generated paired αβTCR sequences to identify and clone Mtb-specific TCRs. Technical limitations prevented us from sequencing TCRs from every T cell, and from cloning and screening TCRs from all the relevant GLIPH2 groups. However, the combined use of GLIPH2 and peptide stimulations allowed us to focus on common CDR3 motifs to estimate the Mtb-specific and bystander T cell responses. While this approach could eliminate cross-reactive TCRs that are Mtb-specific, stringent Mtb specificity requirement for TCRs outweighed the risk of losing a small number of potentially cross-reactive TCRs. We recognize the drawbacks of focusing exclusively on TCRs grouped for their similar motifs, including the possibility of missing “ungrouped” Mtb-specific clonotypes. To address this, we leveraged the published South African datasets to improve GLIPH2 grouping by increasing the numbers of participant and TCRs. Our initial analysis of clonally expanded TCRs contained many that were specific for viral antigens. Like infection, Mtb lysate has adjuvant properties and could have overestimated the lack of recognition of infected macrophages when used as a comparator. Our *ex vivo* assay uses virulent Mtb and a standard macrophage infection model. While the timing of Mtb antigen expression, processing, and presentation could be different *in vivo*, explaining the lack of recognition of infected macrophages by certain TCRs, most clonotypes that responded to MTB300 or lysate also recognized infected cells. Finally, the specificities of many of the TCRs we identified are still yet to be determined. However, this study provides a link between these TCRs and recognition of infected macrophages (or lack thereof), enabling future screens for antigen specificity as technology improves and screens become more efficient.

## Materials and methods

### Study participants

10 healthy participants with LTBI and a median age of 35 years (range 23–68; six male, four female), representing African, Asian, Caucasian, and Hispanic ethnicities, volunteered based on self-identified history of LTBI. LTBI status was determined based on positive results of either a TST of at least 10 mm or an IGRA or both. No LTBI participants had a history of active TB disease or symptoms suggestive of current disease. 8 of 10 participants previously spent time in TB endemic areas, and 5 participants previously received isoniazid or rifampin antibiotic prophylaxis at least 3 years prior to participating. Five individuals had remote TST conversion and had never received antibiotic prophylaxis. Five individuals received the BCG vaccine as infants (>30 years prior to participating). Seven healthy volunteers without LTBI with a median age of 29 years (range 23–53; four male, three female) served as controls, including anonymized leukapheresis products from 10 participants purchased from AllCells. The negative LTBI status of participants was verified through QuantiFERON-TB Gold Plus (Qiagen). The study included both male and female participants, and the findings are expected to be relevant to both sexes. No correlation between participant sex and T cell activation was observed, although sample sizes were limited. All protocols involving human subjects were approved through the Institutional Review Board of University Hospitals Cleveland Medical Center. Informed consent was obtained for all participants. Protocols involving lentiviral transduction of human TCR genes were approved by the Institutional Biosafety Committee of Case Western Reserve University.

### Generation of MDMs

Following blood draw, Ficoll-Paque PLUS (GE Healthcare) was used to underlay diluted whole blood using a Pasteur pipette. Following centrifugation, the PBMC layer was aspirated using a transfer pipette. The PBMCs were then washed with Ca^++^- and Mg^++^-free and pyrogen-free sterile phosphate-buffered saline (hereafter termed PBS; Corning) and counted using a hemocytometer after staining with trypan blue (Life Technologies) to exclude dead cells. Once counted, CD14^+^ monocytes were separated from the rest of the PBMCs by positive immunomagnetic selection using anti-human CD14 microbeads (Miltenyi Biotec), per the manufacturer’s instructions. After CD14 selection, both the positive and negative fractions were counted and separately cryopreserved in freezing media (10% DMSO, 90% FBS) in liquid nitrogen.

CD14^+^ monocytes were thawed and rested overnight in complete RPMI 1640 media (cRPMI) containing 10% FBS, 2 mM of L-glutamine, Na-pyruvate, nonessential amino acids, 0.01 M Hepes buffer, 2-mercaptoethanol, and 0.005 M NaOH (Gibco), as described previously (Gail et al., 2024). The next morning, CD14^+^ cells were plated in either a 96-well plate (50,000 cells/well) or a 24-well plate (250,000 cells/well) and differentiated to macrophages using GM-CSF (25 ng/ml) (PeproTech). Half of media in each well was changed at 3 days with GM-CSF–containing cRPMI. After a total of 6 days, the macrophages were ready for Mtb infection and media from the entire well were exchanged with cRPMI prior to Mtb infection, as described previously (Gail et al., 2023; Gail et al., 2024).

### Bacterial culture and infection

A 1 ml aliquot of Mtb strain H37Rv (NR-13648; BEI Resources) was thawed and diluted in 9 ml of media containing Difco Middlebrook 7H9 broth (BD Diagnostics) supplemented with 10% Middlebrook oleic acid, bovine albumin, dextrose, and catalase (OADC) Growth Supplement (Millipore), 0.2% glycerol, and 0.05% Tween-80, and culture was expanded for use at the mid-log phase over 6 days, as described previously (Gail et al., 2023; Gail et al., 2024). H37Rv was selected since it is a virulent reference strain of Mtb, since lysate was also prepared from H37Rv, and for comparability with the majority of published studies of human macrophage *in vitro* infection models. Cultures were washed and filtered through a 5-μm Millex syringe-driven filter unit (Millipore) to generate a single-cell suspension. The bacterial count was estimated using the OD_600_ of filtered bacteria in cRPMI and added to macrophage wells to target an MOI of 4–5, as described previously (Gail et al., 2023; Gail et al., 2024). After 4 h, wells were washed of extracellular bacteria and incubated in fresh cRPMI overnight. Actual MOIs were enumerated from three to four separate wells of Mtb-infected macrophages as described previously (Gail et al., 2023; Gail et al., 2024; Carpenter et al., 2017).

### CD4 memory T cell coculture assay

CD14^−^ PBMCs were thawed and rested in cRPMI overnight. Immunomagnetic selection was performed per the manufacturer’s instructions using the human memory CD4 T cell isolation kit (Miltenyi Biotec) for negative selection of memory (CD45RA^Lo^) CD4^+^ T cells. autoMACS Rinsing solution with 5% MACS BSA Stock Solution (Miltenyi Biotec) was used to wash cells, hereafter termed “Rinse Buffer.” After selection, CD4^+^ T cells were then added at ∼4:1 ratio to the infected macrophages in 24-well plates. Ultra-LEAF anti-CD40 blocking antibody (clone W17212H) (RRID: AB_2814511) (BioLegend) at 1 μg/ml was included with T cells and infected macrophages for detection of CD40L expression on T cells, as described previously (Gail et al., 2023; Gail et al., 2024). For scRNAseq experiments, anti-CD40 mAb blockade was not performed. Instead, PE-Dazzle-594 anti-CD40L mAb (clone 24–31) (RRID: AB_2566245) (BioLegend) was added to identify CD40L that may become internalized after ligation with CD40, avoiding blockade, as described previously (Musvosvi et al., 2023; Gail et al., 2024). CD4^+^ T cells were cocultured with infected macrophages in cRPMI at 37°C and 5% CO_2_ overnight. A cocktail of anti-MHC-II blocking antibodies (anti-HLA-DR, clone L243 (RRID: AB_396143), anti-HLA-DR, anti-HLA-DP, anti-HLA-DQ, clone Tu39 (RRID: AB_395938), no azide, low endotoxin grade) (BD Biosciences) was added (final concentration of 25 μg/ml, each) to some Mtb-infected samples prior to the addition of T cells as negative controls. Positive controls included stimulation with 1ug/ml staphylococcal enterotoxin B (Toxin Technology, Inc.) or Mtb lysate (for LTBI samples).

### Flow cytometry staining and analysis

After 16 h, T cells were harvested with autoMACS Rinsing Buffer (Miltenyi). For a subset of experiments, the Miltenyi IFNγ Secretion Assay was performed according to the manufacturer’s instructions, followed by viability dye Live-Dead Aqua or Violet (Invitrogen) staining, as described previously (Gail et al., 2024). Immunostaining was performed with fluorescently labeled antibodies and FcR block (BioLegend) diluted in autoMACS running buffer. After 20 min at 4°C, cells were washed and fixed in 1% PFA in PBS for 1 h, followed by removal from the BSL-3 lab. Samples were acquired on LSRFortessa X-20 Cell Analyzer (BD). Fluorescent antibodies used in flow cytometry and sorting experiments included the following: APC (RRID: AB_1727507)- or BUV395 (RRID: AB_2738770)-conjugated anti-human CD69 (FN50), BUV737 anti-CD3 (UCHT1) (RRID: AB_2870081), BV650 anti-CD45RA (HI100) (RRID: AB_2738514), BUV395 anti-CD4 (RPA-T4) (RRID: AB_2738917), and BB515 anti-CD25 (M-A251) (RRID: AB_2739065), from BD Biosciences; and BV785 anti-CD4 (RPA-T4) (RRID: AB_2564382), PE anti-mouse TCRβ (H57-597) (RRID: AB_313431), FITC anti-CD19 (HIB19) (RRID: AB_2564143), and PE-Dazzle-594 anti-CD40L (24–31) (RRID: AB_2566245) from BioLegend.

### Flow sorting of activated T cells for scRNAseq

Nonadherent cells were harvested from T cell–infected MDM coculture, and immunostaining was performed, as described previously (Gail et al., 2024). After 20 min at 4°C, cells were passed through 40-μm filter top tubes (Corning). ∼2 min prior to sorting each sample, 7-AAD viability dye (Invitrogen) was added to each sample. Samples were sorted using the “purity” setting, gating on 7-AAD^lo^ CD4^+^ single cells, and co-expression of CD69 and either CD40L or CD25. Sorting was performed on Sony MA900 Multi-Application Cell Sorter (Sony Biotechnology). Fluorescent antibodies used in flow sorting experiments include FITC anti-human CD4 (clone RPA-T4) (RRID: AB_313074), PE anti-CD25 (clone BC96) (RRID: AB_314276), PE-Dazzle-594 anti-CD40L (clone 24–31) (RRID: AB_2566245), BV605 anti-CD8α (clone RPA-T6) (RRID: AB_2563185), and APC anti-CD69 (clone FN50) (RRID: AB_314845) from BioLegend.

### Exogenous antigen stimulation of CD4^+^ T cells

Exogenous antigens were added to some samples of Mtb-infected MDMs for coculture with autologous CD4^+^ T cells. H37Rv whole-cell lysate (NR-14822; BEI Resources) was added to Mtb-infected MDMs at a final concentration of 10 μg/ml for 24 h. Lysate was washed out with cRPMI prior to the addition of CD4^+^ T cells. For evaluation of T cell responses to MTB300 peptides (Lindestam Arlehamn et al., 2013), MTB300 was diluted to 1 μg/ml in cRPMI, then added to Mtb-infected MDMs ∼10 min prior to adding CD4^+^ T cells in coculture. For each participant, PBMCs were also separately stimulated with either MTB300 or viral and vaccine control peptide megapools to identify TCR clonotypes linked to antigen-specific T cell responses. For Mtb peptide stims, 1 μg/ml of the MTB300 peptide megapool was added to 10 × 10^6^ PBMCs in cRPMI. For control peptide stimulations, another 10 × 10^6^ PBMCs were exposed to a final concentration of 1 μg/ml each of CMV, EBV, *B. pertussis*, and tetanus toxoid peptide megapools (Dan et al., 2016; Lindestam Arlehamn et al., 2016; Carrasco Pro et al., 2015; da Silva Antunes et al., 2017), and 1ug/ml of the SARS-CoV-2 USA-WA1/2020 coronavirus spike (S) protein overlapping peptides (BEI Resources NR-52402). Peptides were added to PBMCs in cRPMI together with anti-CD28/CD49d costimulatory mAbs (BD Biosciences) and anti-CD40 blocking mAb (RRID: AB_2814511) (BioLegend) for 16–18 h. Following stimulation, CD4-positive immunomagnetic selection was performed according to the manufacturer’s instructions using human anti-CD4 microbeads (Miltenyi Biotec), followed by flow sorting of the activated CD4^+^ T cells for downstream scTCRseq.

### Sample processing for scRNAseq

Sequencing of mRNA from single T cells was performed using the 10X Genomics Chromium Next GEM Single Cell 5′ V2 platform (10X Genomics). Activated CD4^+^ were resuspended in 0.04% BSA in PBS and loaded onto the 10X Chromium Controller. RNA was reverse-transcribed, and cDNA was isolated and PCR-amplified according to the 10X Genomics NextGEM 5′ V2 scRNAseq User Guide (Rev D). Quality control and quantification of cDNA and final libraries were performed on either the Agilent fragment analyzer or Agilent 2100 Bioanalyzer (Agilent Technologies). V(D)J amplification and library preparation, and gene expression library construction were performed using 10X Genomics kits according to the User Guide. Paired-end sequencing was performed by the Cleveland Clinic Lerner Research Institute Genomics Core using an Illumina NovaSeq 6000 sequencer (Illumina) according to the 10X Genomics User Guide. Demultiplexed FASTQ files for TCR sequencing and single-cell gene expression (available in Gene Expression Omnibus (GEO) accession number GSE305300) were mapped to the human genome using Cell Ranger Multi V7.1 (10X Genomics) on the 10X Cloud server and the GRCh38.p14 reference genome.

### Single-cell data analysis

Filtered_contig_annotations.csv and clonotypes.csv files from Cell Ranger containing productive, full-length TCR sequences for each sample were used for TCR clonotype analysis. Filtered feature barcode matrices (mRNA data) and contig annotations (CDR3α and β sequences) were linked to individual cell barcodes. Gene expression and TCR annotations were generated for each cell for simultaneous TCR and transcriptomics analysis after quality control and integration were performed using the R-based Seurat package (v 5.1.0) (Satija et al., 2015) with seed 123. High-quality cells and features were selected based on the following parameters: transcript detection in at least three cells, 200 < nFeature < 4,000, and percentage of mitochondrial RNA is <20% per cell. TCR information was added to Seurat objects using the scRepertoire package (Borcherding et al., 2020). TCR clonotypes with identical CDR3α/β sequences, present in more than two cells (based on unique barcodes), were considered “expanded.” Clonotypes with only an individual CDR3α or CDR3β chain were classified as “nonexpanded.” After merging the samples, SCTransform (Hafemeister and Satija, 2019) was used for normalization, the percent mitochondrial and ribosomal counts, and cell-cycle gene scores were regressed out, and TCR genes were removed from the variable feature list using the quietTCRgenes function from the Trex package (Borcherding et al., 2024). Principal component analysis (RunPCA function) was followed by data integration and batch correction with the Harmony algorithm (Korsunsky et al., 2019). A UMAP (via RunUMAP) was calculated using the first 30 dimensions of the Harmony embedding with default parameters except the number of neighbors was set to 50 in addition to the FindNeighbors default parameters. Clustering was done on this shared-nearest neighbor graph using the FindClusters default parameters except the resolution was set to 0.3 using the Louvain algorithm with multilevel refinement. From 19 clusters obtained (from 0 to 18) (Fig. S3 A), clusters 17 (27 cells) and 18 (25 cells) were removed due to low cell numbers, and cluster 16 (830 cells) was removed due to monocytic contamination. A total of 157,462 cells split into 16 clusters were left for the downstream analysis.

### Clonal TCR analysis

The total set of TCR sequences from LTBI vs. non-LTBI participants was compared for TCR clonality/diversity using entropy indices (Shannon and Inverse Simpson) (Stewart et al., 1997). Unique αβTCR sequences (clonotypes) present in ≥2 copies were used to compare the responses to Mtb-infected macrophages ± MTB300 peptides, or infected macrophages ± lysate. A combined list of the unique (and total) TCR clonotypes identified in response to Mtb-infected macrophages + MTB300 peptides was generated from all experiments, which evaluated a response pair (infected macrophages ± MTB300). The list of TCRs was compared with the combined list of TCRs from responses to infected macrophages only, to determine the percentage recognizing infected macrophages (or MTB300 peptides only). The same approach was taken for paired analysis of infected macrophages ± lysate. TCR clonotype diversity was assessed using Normalized Shannon Entropy and Inverse Simpson indices. Prior to diversity calculation, clonotype counts were rarefied to the minimum sequencing depth observed across samples. Each sample underwent 1,000 rounds of rarefaction to estimate median diversity indices and IQR for each sample. Median values were used to represent each individual sample for comparison of indices between groups (LTBI vs. non-LTBI). MAIT Match 1.0 (https://services.healthtech.dtu.dk/services/MAIT_Match-1.0/) was used to evaluate CDR3 sequences for MAIT cell homology. MAIT, iNKT, and GEM T cells were defined as MAIT Match score of 1 or ≥0.97 (for MAIT and MAIT-like TCRs, respectively), TRAV10;TRAJ18 and “CVVSDRGSTLGRLYF” CDR3α sequence (for iNKT cells), and TRAV1-2;TRAJ09 and “CAV[RL].TGGFKTIF” CDR3α sequence (for GEM cells), respectively, as described previously (Ogongo et al., 2019; Musvosvi et al., 2023; Van Rhijn et al., 2013), with “[]” containing the permitted amino acid and “.” denoting any amino acid.

### GLIPH2 and TCRdist3 TCR analysis

A list of all HLA-II alleles for each participant, and a combined list of all CDR3β, CDR3α, Vβ, and Jβ genes, and a number of clones, for expanded TCRs (≥2 copies) from each participant and experimental condition, were generated. In cases where two CDR3β sequences were identified in a cell, they were separated and each linked to the same CDR3α, V, and J genes for compatibility with GLIPH2 (RRID: SCR_025690). HLA typing was performed by the University Hospitals of Cleveland Histocompatibility and Immunogenetics Laboratory. GLIPH2 analysis was performed as described previously (Huang et al., 2020) using BLOSUM62 restriction for interchangeable amino acids. Using the jvenn (RRID: SCR_016343) (Bardou et al., 2014) Venn diagram builder, GLIPH2 groups containing TCRs linked to a response to control peptides were separated from analysis. TCR sequences within GLIPH2 groups were cross-referenced with TCRs annotated in the IEDB (RRID: SCR_006604), and we removed entire GLIPH2 groups that contained any CDR3βs that contained ≥97% homology with TCRs previously annotated as specific for viral antigens in other studies.

To increase robustness of GLIPH2 groups, a TCR dataset containing >100 participants from 3 published studies, from Musvosvi et al. (2023), and TCRs from Cleveland participants were added, followed by reanalysis using GLIPH2. In addition to the statistical criteria above, only GLIPH2 groups containing TCRs from ≥3 participants and an HLA association score <0.1 were evaluated. Sequence logo plots of the CDR3β and CDR3α chains were generated using WebLogo3 (RRID: SCR_010236) (Crooks et al., 2004). GLIPH2 groups with consistent CDR3α homology (differing by ≤2 amino acids) were identified for transcriptomics mapping. TCRdist3 meta-clonotypes were found using the default settings for sample distance calculation and background generation. For calculating a joint distance matrix, a radius of 100 was used (Mayer-Blackwell et al., 2021). The Fisher score (GLIPH2), regex score (TCRdist3), and number of donors represented in each group were used to compare the two algorithms. The 85 TCRs from 29 GLIPH2 groups from the combined dataset, which were estimated to be Mtb-specific and passed statistical thresholds, were also evaluated for grouping into TCRdist3 meta-clonotypes. Sankey plots were used to compare grouping of these TCRs by either algorithm. Chi-square testing was used to compare all TCRs grouped by either algorithm as indicated.

### Bulk TCRβ deep sequencing

Unstimulated CD4^+^ T cells were isolated by immunomagnetic positive selection from 10 × 10^6^ PBMCs from each participant using human anti-CD4 microbeads (Miltenyi Biotec). Genomic DNA was isolated using QIAamp DNA Mini Kit (Qiagen) according to the manufacturer’s instructions. Ultra-deep-level TCRβ sequencing was performed by Adaptive Biotechnologies. Data were downloaded from the ImmunoSeq Analyzer (Adaptive) and used to estimate the natural circulating frequencies of TCRs. TCRβ sequences were identified in the scTCRseq analysis of CD4^+^ T cell responses to Mtb-infected macrophages, cross-referenced to CDR3β sequences in bulk TCRβ deep sequencing data from PBMCs, which were enumerated using Microsoft Excel and graphed in GraphPad Prism.

### Cloning of TCRs

TCR sequence transfection methods were adapted from Dezfulian et al. (2023). Nucleotide sequences for selected TCRs were synthesized (Twist Biosciences) and inserted into pDONR221 Entry Vector (Invitrogen). All TCRs were engineered as hybrid TCRs, in which the entire human α and β V(D)J regions were preserved, and these nucleotides were grafted onto mutant mouse α and β constant regions, respectively, and cloned into the pDONR221 Entry vector as described previously (Dezfulian et al., 2023). Mutant mouse constant regions were used to increase TCR abundance by removing a degron within the TCRα chain, to enhance TCR association with CD3 for more efficient T cell signaling, and to improve specific pairing and identification of the cloned TCRs. The codon-optimized nucleotide sequence for hybrid αβTCR sequences was synthesized (Twist Biosciences) as a single polyprotein separated by a 2A self-cleaving peptide sequence (TCRβ-P2A-TCRα) and subsequently cloned into the pDONR22 entry vector and then transferred into the pHAGE-EF1:DEST-PGK:CD4 destination vector provided by Dr. Mohammad Haj Dezfullian (University of Pennsylvania Perelman School of Medicine, Philadelphia, PA, USA), using LR Clonase (Invitrogen) according to the Gateway cloning methodology (Invitrogen) and as described previously (Dezfulian et al., 2023), and used to transduce 2T1R-competent cells (Invitrogen). Selection of target cells was performed using ampicillin (100 μg/ml) (Gibco).

Lentivirus was produced through the cotransfection of packing plasmids (Tat, Rev, Gag-Pol, and VSV-G) (Addgene) and destination vector into the HEK293T cell line (Takara Biosciences), using jetPRIME (Polyplus). Lentiviral supernatants were collected 48 h after HEK293T transfection, filtered with a 0.45 μm filter (Millipore), and concentrated using 100k Macrosep Advance Centrifugal Devices (Pall Corp.).

Transduction using lentiviral supernatants was performed in the SKW-3 cell line (RRID: CVCL_2197) (Schrieb Leibniz-Institut DSMZ, Germany). Concentrated lentivirus was added to SKW-3 cells suspended in cRPMI at 1 × 10^6^ cells/ml, then incubated for 72 h at 37°C in 5% CO_2_. Transduced cells were washed, then positively selected using immunomagnetic selection using biotinylated or PE-conjugated anti-mouse TCR-β, followed by anti-PE microbeads (Miltenyi Biotec) according to the manufacturer’s instructions. Antigen screening was performed either before or after selection using the SKW-3 cell line transduced with the TCR of interest.

### Generating donor-specific APC cell lines

Autologous EBV-transformed B cell lines were prepared according to the ATCC Lymphocyte transformation protocol (https://www.atcc.org). Briefly, irradiated MRC-5 feeder (ATCC-55X) (RRID: CVCL_RB14) (ATCC) cells were plated in flasks. 3 days later, B cells were isolated from PBMCs by immunomagnetic negative selection using human anti-CD3 and anti-CD14 microbeads (Miltenyi Biotec). The negative fraction was co-incubated with the MRC-5 feeder cells and transformed by adding human gammaherpesvirus (HHV-4; ATCC-VR 1492). After 14–21 days, when signs of successful transformation are visible, cells were transferred to larger flasks, expanded in culture, and then cryopreserved for future use.

### Antigen and infected macrophage screening of cloned TCRs

TCR-transduced SKW-3 cells were expanded and then cocultured 1:1 with donor-matched EBV-transformed B cells loaded with each of the fifteen 20-peptide subpools of MTB300, at final concentrations of 1 μg/ml, overnight at 37°C in 5% CO_2_. DMSO (Sigma-Aldrich), MTB300 (1 μg/ml), and CD3/CD28 Human T-activator Dynabeads (Gibco) were used as controls. Immunostaining with anti-CD4, CD19, TCRβ, and CD69 along with Live/Dead was performed, followed by fixation in 1% PFA/PBS. The portion of TCRβ+ cells expressing CD69 was enumerated by flow cytometry. Upon observing reactivity to a subpool, the assay was repeated using each of 20 individual peptides (at 1 μg/ml) for the subpool, in addition to controls, to identify the peptide that led to CD69 expression. Cognate peptides for each TCR that responded to individual peptides were confirmed by mass spectroscopy (Cleveland Clinic Lerner Research Institute Proteomics and Metabolomics Core Facility) of individual peptide stocks or by stimulation with newly synthesized peptides. Cognate peptides for CFP10-specific TCRs were confirmed using 15 or 16-mer peptides from an overlapping peptide array (BEI Resources NR-50712).

Validation of recognition of infected macrophages was performed for each TCR-transduced SKW-3 cell line. One day after Mtb infection of macrophages, each TCR-transduced SKW-3 cell line was added in coculture (1:1) for 16–18 h, using the same methodology as with primary CD4^+^ T cells (see above). Autologous CD14^+^ monocytes, or those derived from anonymized PBMCs, purchased from AllCells and matched to established HLA restriction for each TCR, were used to generate MDMs. Activation in response to infected macrophages was estimated by CD69 expression by flow cytometry.

### Single-cell transcriptomics, pathway, and cell interaction analyses

Gene expression visualization was performed using the Nebulosa package (v 1.16.0) (Alquicira-Hernandez and Powell, 2021). To identify cluster-defining genes, the FindConservedMarkers function was run on the integrated dataset with the following parameters: min.pct = 0.25 and logfc.threshold = 0.25; the results were arranged according to average log_2_FC, and the top 5 genes for each cluster were visualized in a dotplot in Fig. 6 C using the scCustomize package (v 2.1.2). Single-cell trajectory analysis and projection were done using the Monocle3 package (Cao et al., 2019). The list of DEGs for each cluster was determined from the integrated dataset and running FindAllMarkers function with the following parameters: min.pct = 0.25, logfc.threshold = 0, test.use = “MAST,” recorrect_umi = FALSE. Genes upregulated in selected clusters were visualized with the dittoSeq package (v 1.19.0) (Bunis et al., 2020). Reactome pathway gene set enrichment analysis (Milacic et al., 2023) was performed using the clusterProfiler package (v 4.14.4) (Xu et al., 2024). Modeling of intercellular communication (i.e., ligand activity analysis, target gene prediction, and ligand–receptor interactions) was performed using the nichenetr package (v 2.2.0) (Browaeys et al., 2020).

### Statistics

Flow cytometry was analyzed using FlowJo v10 (BD Biosciences). Graphs and statistical comparisons were performed on Prism v10 (GraphPad) using tests specified in figure legends. Grouped data were compared using nonparametric tests (Wilcoxon matched-pairs signed rank test). Data were tested for normality using the Shapiro–Wilk or Kolmogorov–Smirnov tests in Prism V10. If normally distributed, experiments with two conditions were compared using a paired or unpaired *t* test with Welch’s correction. Experiments with multiple comparisons were compared using a Welch one-way ANOVA and Dunnett’s T3 post hoc test corrected for multiple comparisons. Comparisons of TCRs grouped by GLIPH2 and TCRdist3 were performed using a chi-square analysis and Cramer’s V post hoc test. Two-tailed P values <0.05 were considered significant. For single-cell transcriptomics analysis, per-cluster quantification of cell counts, clonal sizes, and CDR3 motifs was done using the R package Speckle (v 1.7.0) (Phipson et al., 2022). GLIPH2 groups considered for further analysis contained either >2 unique CDR3β sequences, TCR Vβ gene homology score (P < 0.05), CDR3β length distribution score (P < 0.05), and a Fisher exact score (P < 0.05) for distinct CDR3β motifs from the V1 reference set of TCRs, or HLA association score for GLIPH2 analysis of combined TCR datasets (one-sided P < 0.05), as reported previously (Musvosvi et al., 2023). Differential gene expression analyses were performed using the Seurat function FindAllMarkers to identify significantly expressed genes at P < 0.05.

### Online supplemental material

Fig. S1 shows the co-expression of CD69 and CD25 among memory CD4^+^ T cells in response to infected macrophages ± MTB300 or lysate using flow cytometry, and the gating strategy for flow sorting–activated CD4^+^ T cells. Fig. S2 shows the distribution of GLIPH2 groups linked to viral or vaccine control responses and the proportions of HLA allelic overlap between Cleveland and South African participants. Fig. S3 shows quality control metrics for cell clusters after batch correction. Fig. S4 shows the mapping of TCR clonotypes with inconsistent CDR3α chain homology onto Louvain clusters of single-cell transcriptomics data. Fig. S5 shows the intercellular communication between Mtb-specific TCR clonotypes and other memory CD4^+^ T cells. Data S1 contains the TCRs within each GLIPH2 group in Fig. 4 estimated to be Mtb-specific. Data S2 contains the TCRs within each GLIPH2 group in Fig. 4 estimated to be specific for other pathogens. Data S3 lists CDR3β sequences from Mtb-specific GLIPH2 groups also grouped by TCRdist3 in Fig.4. Data S4 lists TCR sequences cloned into SKW-3 cells for screening peptides and infected macrophage responses in Figs. 5 and 6. Data S5 lists the MTB300 peptides divided into the 15 subpools used for peptide screening in Fig. 5.

## Supplementary Material

Data S1 contains the TCRs within each GLIPH2 group in Fig. 4 estimated to be Mtb-specific.

Data S2 contains the TCRs within each GLIPH2 group in Fig. 4 estimated to be specific for other pathogens.

Data S3 lists CDR3β sequences from Mtb-specific GLIPH2 groups also grouped by TCRdist3 in Fig.4.

Data S4 lists TCR sequences cloned into SKW-3 cells for screening peptides and infected macrophage responses in Figs. 5 and 6.

Data S5 lists the MTB300 peptides divided into the 15 subpools used for peptide screening in Fig. 5.

## Data Availability

The original code reported in this paper is publicly available through GitHub (https://github.com/carpenter-lab/StetsenkoGail2025.git). Raw single-cell sequencing data used in Figs. 7, 8, and 9 are publicly available through the NCBI GEO accession number GSE305300. Cell Ranger gene mapping matrices, TCR sequences, and GLIPH2 outputs used for individual figures are publicly available through the Dryad repository (https://doi.org/10.5061/dryad.vmcvdnd53). Source data underlying Figs. 4, 5, and 6 are available as Supplemental Data in the published article. Other data will be provided by the corresponding author upon reasonable request.
